# Preclinical validation and phase I trial of 4-hydroxysalicylanilide, targeting ribonucleotide reductase mediated dNTP synthesis in multiple myeloma

**DOI:** 10.1186/s12929-022-00813-2

**Published:** 2022-05-12

**Authors:** Yongsheng Xie, Yingcong Wang, Zhijian Xu, Yumeng Lu, Dongliang Song, Lu Gao, Dandan Yu, Bo Li, Gege Chen, Hui Zhang, Qilin Feng, Yong Zhang, Ke Hu, Cheng Huang, Yu Peng, Xiaosong Wu, Zhiyong Mao, Jimin Shao, Weiliang Zhu, Jumei Shi

**Affiliations:** 1 Department of Hematology, Shanghai Tenth People’s Hospital, Tongji University School of Medicine, 301 YanChang Road, Shanghai, 200072 China; 2 CAS Key Laboratory of Receptor Research; State Key Laboratory of Drug Research; Drug Discovery and Design Center, Shanghai Institute of Materia Medica, Chinese Academy of Sciences, 555 Zuchongzhi Road, Shanghai, 201203 China; 3 Department of Pathology and Pathophysiology, Cancer Institute of the Second Affiliated Hospital, Zhejiang University School of Medicine, Zhejiang University Cancer Center, 866 Yuhangtang Road, Hangzhou, 310058 Zhejiang China; 4 Clinical and Translational Research Center of Shanghai First Maternity and Infant Hospital, Shanghai Key Laboratory of Signaling and Disease Research, Frontier Science Center for Stem Cell Research, School of Life Sciences and Technology, Tongji University, Shanghai, 200092 China

**Keywords:** 4-Hydroxysalicylanilid, Ribonucleotide reductase, Deoxyribonucleotides, DNA damage repair, Multiple myeloma

## Abstract

**Background:**

Aberrant DNA repair pathways contribute to malignant transformation or disease progression and the acquisition of drug resistance in multiple myeloma (MM); therefore, these pathways could be therapeutically exploited. Ribonucleotide reductase (RNR) is the rate-limiting enzyme for the biosynthesis of deoxyribonucleotides (dNTPs), which are essential for DNA replication and DNA damage repair. In this study, we explored the efficacy of the novel RNR inhibitor, 4-hydroxysalicylanilide (HDS), in myeloma cells and xenograft model. In addition, we assessed the clinical activity and safety of HDS in patients with MM.

**Methods:**

We applied bioinformatic, genetic, and pharmacological approaches to demonstrate that HDS was an RNR inhibitor that directly bound to RNR subunit M2 (RRM2). The activity of HDS alone or in synergy with standard treatments was evaluated in vitro and in vivo. We also initiated a phase I clinical trial of single-agent HDS in MM patients (ClinicalTrials.gov: NCT03670173) to assess safety and efficacy.

**Results:**

HDS inhibited the activity of RNR by directly targeting RRM2. HDS decreased the RNR-mediated dNTP synthesis and concomitantly inhibited DNA damage repair, resulting in the accumulation of endogenous unrepaired DNA double-strand breaks (DSBs), thus inhibiting MM cell proliferation and inducing apoptosis. Moreover, HDS overcame the protective effects of IL-6, IGF-1 and bone marrow stromal cells (BMSCs) on MM cells. HDS prolonged survival in a MM xenograft model and induced synergistic anti-myeloma activity in combination with melphalan and bortezomib. HDS also showed a favorable safety profile and demonstrated clinical activity against MM.

**Conclusions:**

Our study provides a rationale for the clinical evaluation of HDS as an anti-myeloma agent, either alone or in combination with standard treatments for MM.

*Trial registration*: ClinicalTrials.gov, NCT03670173, Registered 12 September 2018.

**Supplementary information:**

The online version contains supplementary material available at 10.1186/s12929-022-00813-2.

## Background

Multiple myeloma (MM) is a B-cell malignant disorder characterized by clonal proliferation of plasma cells in the bone marrow (BM) [1, 2]. The average survival of MM patients has been improved significantly over the past decade, in line with the emergence of available treatments including proteasome inhibitors [3, 4]. Unfortunately, the median survival is still only 6–7 years [3], making MM an incurable disease with drug resistance.

RNR is an antineoplastic target that manifests therapeutic benefits in many types of cancers [5], and its catalytic activity requires both RRM1 and RRM2 homodimers [6]. RNR catalyzes the rate-limiting step in the *de novo* synthesis of deoxyribonucleoside diphosphates (dNDPs) to provide dNTPs, which are essential for DNA synthesis [7]. Levels of RRM1 subunit remain constant throughout the cell cycle because of its long half-life, whereas RRM2 levels fluctuate throughout the cell cycle, being lowest in G0/G1 and peaking in the S phase [7, 8]. Thereby, RRM2 levels control the cell cycle-dependent activity of RNR [9]. Neoplastic cells require large amounts of dNTPs to support uncontrolled cell proliferation, and RNR levels are accordingly markedly elevated in cancers [7, 10]. Accumulating evidence has shown that dysregulated RRM2 protein levels occur in several tumors, including glioblastoma, prostate, breast, liver, and colorectal cancers [11–15]. The elevated RRM2 levels are accompanied by increased RNR-mediated dNTP production, which in turn fuels DNA synthesis during DNA damage repair and leads to increased genomic instability [16]. RRM2 is identified as one of the major chromosomal instability genes significantly related to drug resistance, rapid relapse, and poor prognosis of MM [17]. RRM2 has therefore been identified as an antineoplastic target with demonstrated therapeutic benefits in many cancers, including hematologic malignancies [7, 10]. However, there are disadvantages including side effects and low efficacy in clinical RNR inhibitors currently [18].

4-Hydroxysalicylanilide (HDS) is a chemically synthesized substance that is also found in nature. In clinical practice, it is used as a traditional cholagogue for promoting bile drainage and protecting liver function [19, 20]. Besides, HDS is reported to efficiently inhibit the replication of hepatitis B virus with limited toxicity in vitro and in vivo [20, 21]. Moreover, active metabolites of HDS are reported to induce hepatocellular carcinoma cell apoptosis by activating p53-related pathways [22]. Therefore, given the clinical advantages of HDS and its potential effect on inducing cell apoptosis, we wondered whether HDS could play a role in MM. In the present study, we conducted experiments to clarify the specific interaction between HDS and RRM2, and showed that HDS could target RRM2 in MM cells. We have patented the application of HDS as an anti-myeloma drug with the U.S. Patent and Trademark Office, and initiated a phase I clinical trial of single-agent HDS in MM patients. Here we demonstrate the anti-MM efficacy of HDS in vitro and in vivo, as well as in clinical patients. These findings provide the proof-of-concept for clinical evaluation of HDS as a potential anti-MM agent for MM therapy, alone or in combination.

## Materials and methods

### Cells

Cell lines were obtained from American Type Culture Collection (ATCC) or kindly provided by sources indicated in Additional file 1: Supplemental Methods. Primary BM cells were donated by healthy donors and MM patients with written consent and approved by the Review Board and Ethics Committee of Shanghai Tenth People’s Hospital. Details are available in 1: Supplemental Methods.

### Nuclear magnetic resonance (NMR) spectroscopy

Ligand observed T1ρ and saturation transfer difference (STD) NMR experiments were applied to investigate ligand-protein interactions. All NMR spectra were acquired at 25 °C on a 600 MHz Bruker Avance III spectrometer equipped with a cryogenically-cooled probe (Bruker Biospin, Ettlingen, Germany). Samples containing 200 µM HDS alone or in the presence of 5 µM RRM2 protein were dissolved in Tris-HCl buffer (50 mM Tris-HCl, 100 mM NaCl, pH 7.4, 5% dimethylsulfoxide (DMSO), 95% D_2_O) prior to NMR data acquisition. T1ρ spectra were recorded by using the pulse sequence of solvent-suppressed 1D ^1^H CPMG (cpmgPr1d). The 90° pulse length was adjusted to about 11.82 µs. A total of four dummy scans and 64 free induction decays were collected into 13 K acquisition points, covering a spectral width of 8 kHz (13.3 ppm) and giving an acquisition time of 3 s. STD data was acquired using four dummy scans and a relaxation delay of 3 s, followed by a 40 dB pulsed irradiation at a frequency of − 1.0 ppm or 33 ppm, alternatively. The total acquisition time for the STD spectrum was 21 min with 128 free induction decays.

### Surface plasmon resonance (SPR)

Biacore T2000 (GE Healthcare) was used to perform SPR experiments. Recombinant RRM1 or RRM2 were prepared in 10 mM sodium acetate (pH = 5.0) and then immobilized on a CM5 sensor chip via amine-coupling procedure at 25 °C. The rest binding sites of the sensor chip were blocked by ethanolamine. For HDS-RRM2 or HDS-RRM1 binding analysis, a series dilution of HDS was prepared in PBS buffer (10 mmol/L HEPES pH = 7.4, 150 mmol/L NaCl, 3 mmol/L EDTA), and was flowed over the chip at the rate of 30 mL/min. The association and dissociation time was set at 120 and 150 s, respectively. Data analysis was finished via the state model of T2000 evaluation software (GE Healthcare).

### Cellular thermal shift assay (CETSA)

CETSA was performed as described previously [23]. Briefly, cells were incubated with HDS, HU, gemcitabine, or DMSO for 6 h. Then cultured cells were harvested, washed with ice-cold PBS and diluted with cell lysis buffer (25 mM Tris-HCl (pH 7.5) and 10 mM MgCl_2_) supplemented with complete protease inhibitor cocktail. Cells were aliquoted into PCR tubes (100 µL each) and then incubated at 25, 55, 64, 67, 70, or 73 °C for 4 min. The cell suspension was frozen and thawed three times in liquid nitrogen, and centrifuged at 20,000×*g* for 20 min at 4 °C. The supernatants were quantified and analyzed by western blotting.

### RNR activity measurement

The RNR activity was measured as previously described with a modification that the quantification used a liquid chromatography/tandem mass spectrometry (LC–MS/MS) system [21, 24]. MM cells were treated with different concentrations of HDS for the indicated time. MM cells were collected, washed with cold PBS, lysed with a low salt homogenization buffer (2 mM dl-Dithiothreitol (DTT) and 10 mM HEPES, pH 7.2), and passed through a 27-gauge needle 20 times on ice. Then the same volume of high salt buffer (1 M HEPES, pH 7.2, with 2 mM DTT) was added. The cell suspension was passed through the needle 20 times on ice again. Cell debris was centrifuged at 16,000×*g* for 20 min at 4 °C. Supernatants were separated and passed through a spin column (Sephadex G25). 100 µL reaction mixture was prepared containing 2 mM ATP, 4 mM magnesium acetate, 4 mM DTT, 50 mM HEPES (pH 7.2), 0.1 mM CDP, and a certain amount of cell extracts. Then the reaction mixture was incubated at 37 °C for 20 min. The amount of CDP and dCDP formed in the reaction mixture was quantified utilizing an LC–MS/MS system, and then the RNR activity was calculated.

## dNTP pool assay

Intracellular dNTPs were extracted and quantified using previously described methods [16, 17]. Briefly, cells were collected, washed with PBS, deproteinized by the addition of 70% methanol, vortex for 20 s, incubated at − 20 °C for 30 min, sonicated for 15 min in an ice bath and centrifuged. Supernatants were separated and dried under a stream of nitrogen. The residues were resuspended with water containing 10% acetonitrile and centrifuged again. 5 µL aliquot of the resulting supernatants was then injected into an LC–MS/MS system for dNTP measurements.

### Lentivirus packaging and cell transfection

In order to construct RRM1 and RRM2 knockdown and overexpression cell lines, 293 T packaging cell lines were used for lentiviral transfection. To generate lentiviral particles, 293 T cells were transiently transfected with vector DNA, along with the packaging constructs psPAX2 (#12,260, Addgene), pMD2.G (#12,259, Addgene), and the target plasmid using Lipofectamine 3000 (Thermo Fisher Scientific, Waltham, MA, USA) according to the manufacturer’s protocol. After culturing for 48 h, virus-containing supernatants were collected and filtered through a 0.45 μm filter. Myeloma cell lines were infected with a suspension of virus-containing supernatant with 6 µg/mL polybrene and incubated for 24 h at 37 °C. Stable cells were selected by 2 µg/mL puromycin for 5 days.

## In vivo animal experiments

MM cells were inoculated subcutaneously into the right flank of nude mice. All animal studies were approved by the Review Board and Ethics Committee of Shanghai Tenth People’s Hospital (ID: SYXK 2011-0111). Details are available in 1: Supplemental Methods.

### HDS clinical trial

#### Patients

Key inclusion and exclusion criteria are available in Additional file 1: Supplemental Methods. The patient study was registered at clinicaltrials.gov under number NCT03670173 and was approved by the Review Board and Ethics Committee of Shanghai Tenth People’s Hospital (ID: SHSY-IEC-4.0/17-33/01). Informed consent was obtained from all study participants.

#### Patient treatment

Participants were initially given oral capsules with 0.5 g HDS tid daily for two weeks. Subsequently, patients were orally administered with 0.75 g HDS tid daily for two weeks. After the four-week dose-escalation phase, patients received a maintenance dose of 1.0 g HDS tid. One course of HDS treatment lasts four weeks. Response will be assessed at the end of each treatment course, and patients who are assessed for PD (progression of disease) at the end of each course will receive salvage treatment, such as a combined treatment of HDS and dexamethasone or the VCD (bortezomib, cyclophosphamide and dexamethasone) regimen.

#### Evaluation of patient response and toxicity

Evaluation of response was performed every 4 weeks. Criteria for response and progression were based on International Myeloma Working Group Uniform Response Criteria for Multiple Myeloma. The response was evaluated overall cycles of treatment and required confirmation on two consecutive evaluations. Safety and tolerability were assessed according to the adult Common Terminology Criteria for Adverse Events (CTCAEv.4.0) at every visit.

### Statistical analyses

Statistical significance was determined using the student’s t-test for two samples, or by one-way analysis of variance for multiple samples, using SPSS v20.0 software. The survival time was calculated using the Kaplan–Meier method and compared by log-rank test.

## Results

### HDS exerted anti-MM activity depending on RRM2

**Fig. 1:**
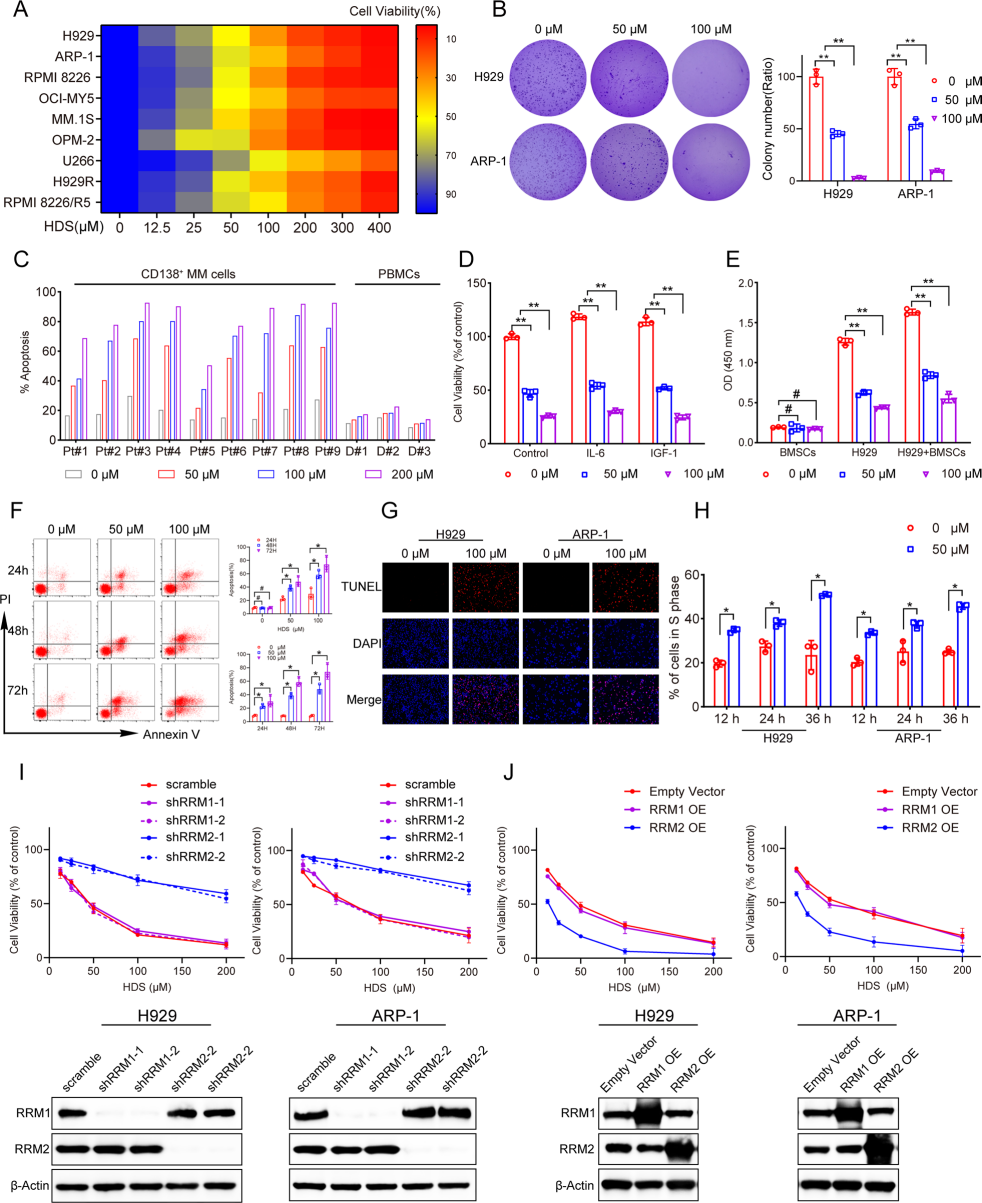
Cytotoxic activity of HDS against MM cells. ** A** Indicated MM cell lines were treated with vehicle or HDS (12.5, 25, 50, 100, 200, 300, 400 µM) for 72 h. Then the cell viability was determined by CCK-8 assay. Cell viability data are presented as the means of 3 independent experiments in a Heatmap. **B** Cell clone colonies formed by the H929 and ARP-1 cells treated with HDS (0, 50 and 100 µM); the colonies in each well were quantified. **C** Primary CD138^+^ MM cells from patients (Pt#1–Pt#9) and normal PBMCs from healthy donors (D#1–D#3) were exposed to HDS with the indicated concentrations for 72 h and then apoptosis was analyzed. Pt represents patient. D represents healthy donor. **D** H929 cells were treated with indicated concentrations of HDS alone or in the presence of IL-6 or IGF-1 for 72 h. Cell viability was determined by CCK-8 assay. **E** H929 cells treated with different concentrations of HDS (0, 50 and 100 µM) were cultured with or without BMSCs for 72 h, and cell growth was assessed using CCK-8 assay. **F** H929 cells were exposed to 0, 50 and 100 µM HDS for indicated time (24, 48 and 72 h). Cell apoptosis was determined by Annexin V/PI staining. Representative results of triplicate experiments were shown. **G** Representative fluorescent images of typical apoptotic cells evaluated by TUNEL staining (red) after 100 µM HDS treatment for 24 h. DAPI was used as a nuclear stain (blue). **H** Cell cycle analysis was performed using flow cytometry. Percentages showed cell population in S-phase. **I** The viability of H929 and ARP-1 cells transfected with scramble or corresponding shRNA plasmids with HDS treatment (0, 12.5, 25, 50, 100 and 200 µM, 72 h) were analyzed by a CCK-8 assay. Western blots showed RRM1 and RRM2 expressions in H929 and ARP-1 cells after transfected with RRM1 or RRM2 shRNA. Notably, the cell viability of RRM1-shRNA transfected cells and scramble-transfected cells were not significantly different at the corresponding HDS concentration. **J** CCK-8 assay was performed on RRM1-OE and RRM2-OE cells or empty vector-transfected cells. Western blots showed RRM1 and RRM2 overexpression in H929 and ARP-1 cells after transfected with RRM1 OE or RRM2 OE vectors. Empty vector-transfected cells served as controls. Data are presented as the means ± SD of 3 independent experiments. *P < 0.05; **P < 0.01; #, not significant

We evaluated the anti-MM activity of HDS in MM cell lines (p53 wild-type: H929 and MM.1 S; p53 mutant: RPMI 8226, OCI-MY5, OPM2 and U266; p53 null: ARP-1) and two bortezomib-resistant MM cell lines (H929R and RPMI 8226/R5). HDS treatment resulted in dose- and time-dependent growth inhibition in all tested cell lines, regardless of p53 status (Fig. 1A, Additional file 1: Fig. S1A, B). Half maximal inhibitory concentration values for these cell lines were determined as follows: 60.27 µM (H929), 71.99 µM (ARP-1), 60.95 µM (RPMI 8226), 73.21 µM (OCI-MY5), 54.0 µM (MM.1 S), 47.06 µM (OPM2), 87.46 µM (U266), 78.63 µM (H929R), and 80.15 µM (RPMI 8226/R5). HDS treatment reduced the colony-forming abilities of MM cells, indicating that it inhibited cell proliferation (Fig. 1B). Similar results were obtained in primary CD138^+^ MM cells. In detail, patients 1–4 who donated bone marrow were newly diagnosed. Patients 5–9 had received a median of 2.6 prior regimens (Additional file 1: Table 1) and were refractory to the effects of bortezomib. CD138^+^ MM cell viability (measured by Annexin V/PI staining) was affected by HDS in a dose-dependent manner (Fig. 1C); however, HDS had no significant cytotoxic effect on normal peripheral blood mononuclear cells (PBMCs) (Fig. 1C). There was thus a significant difference in drug effects between malignant and normal cells.

BMSCs and cytokines IL-6 and IGF-1 in the BM have been shown to promote MM cell proliferation, migration, and drug resistance [25, 26]. Although BMSCs and cytokines increased MM cell viability, HDS still exerted a cytotoxic effect on MM cells (Fig. 1D, E; Additional file 1: Fig. S1C, D). These data suggest that HDS overcomes the cytoprotective effects of BMSCs and cytokines. We next examined the effects of HDS on apoptosis in MM cells by Annexin V/PI staining. Treatment with increasing doses of HDS increased apoptosis in MM cells in time- and dose-dependent manners (Fig. 1F; Additional file 1: Fig. S1E). We confirmed that HDS promoted MM cell apoptosis by detecting the binding of terminal deoxynucleotidyl transferase dUTP nick and labeling (TUNEL) reagent to DNA fragments, a typical morphological characteristic of apoptotic cells (Fig. 1G). Western blot analysis showed that HDS treatment provoked cleavage of caspase-3, -8, and -9, further confirming its apoptotic effect (Additional file 1: Fig. S1F). Notably, the pan-caspase inhibitor Z-VAD-FMK partially blocked the apoptosis caused by HDS (Additional file 1: Fig. S1G), suggesting that HDS-induced apoptosis was at least partly caspase-dependent. We also examined the effect of HDS on MM cell cycle progression, and showed that HDS induced S-phase arrest in MM cells (Fig. 1H).

HDS was shown to target RRM2 using the virtual docking analysis technique [21]. To determine if the anti-myeloma activity of HDS depended on RRM2, we established stable RRM1- and RRM2-knockdown and overexpression MM cell lines. Treatment of RRM2-knockdown cells with HDS led to the loss of sensitivity compared with scrambled control-treated cells, while RRM1-knockdown and scrambled control cells were both sensitive to HDS (Fig. 1I). At the same time, overexpression of RRM2, but not RRM1, increased sensitivity to HDS (Fig. 1J). This indicated that the observed HDS-induced cytotoxicity was related to the RRM2-dependent mechanism.

### HDS targeted RRM2 directly and inhibited intracellular RNR activity in MM cells

**Fig. 2:**
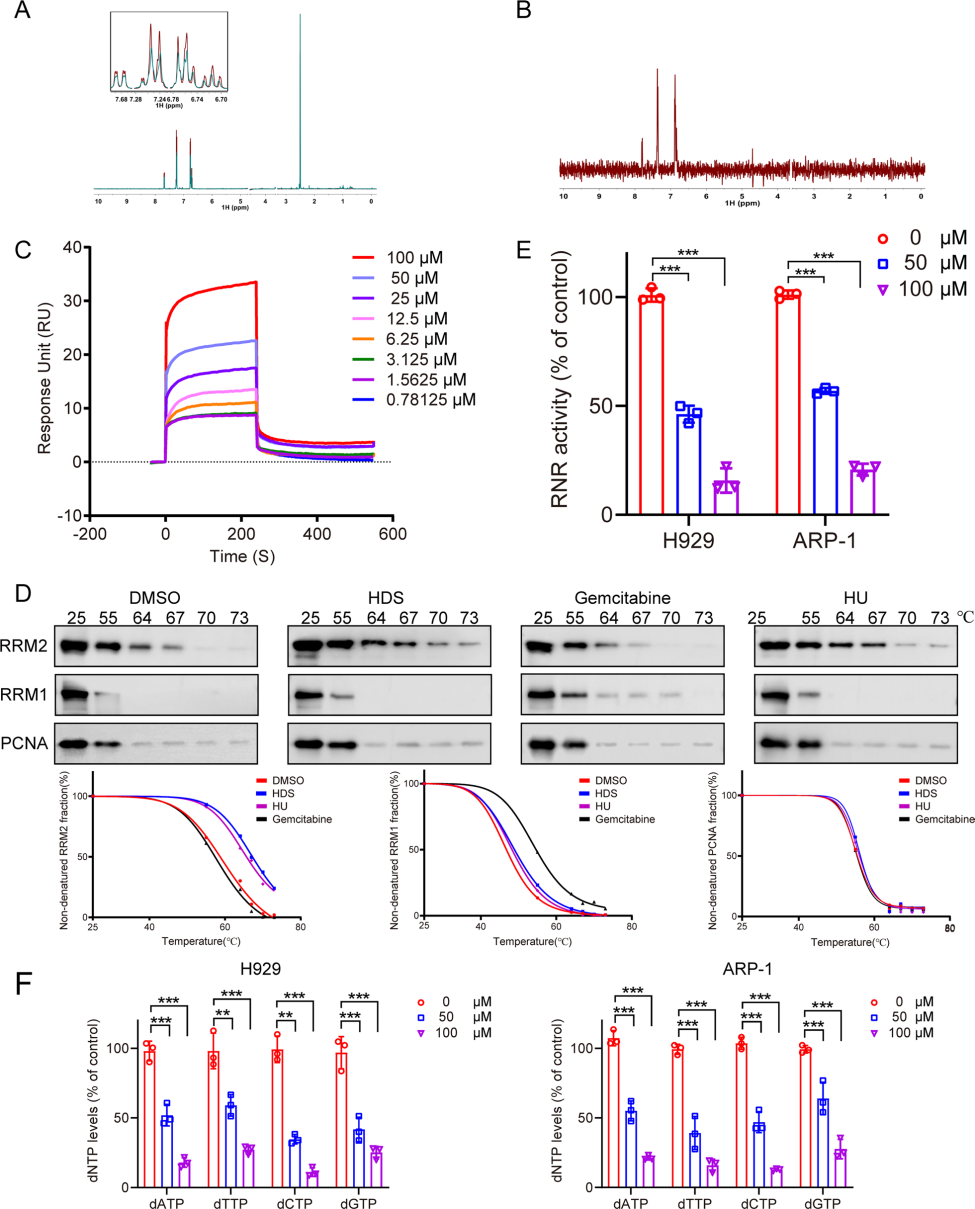
HDS interacted with RRM2 and inhibited RNR activity. ** A** T1ρ spectra acquired by using 200 µM HDS solely (colored in red) and 200 µM HDS in the presence of 5 µM RRM2 protein (colored in green) are presented. **B** STD spectrum acquired by using 200 µM HDS in the presence of 5 µM RRM2 protein (colored in red) is presented. **C** SPR biosensor was used to detect the binding of HDS to RRM2. Representative sensorgrams of the interaction of 0.78125 to 100.0 µM HDS with 200 µg/mL RRM2. **D** Cellular thermal shift assay to examine interactions of compounds (100 µM HDS, 0.5 µM gemcitabine, or 500 µM HU) with RRM1 and RRM2. Lower panel is the charts of percentages of non-denatured protein fraction. **E** MM cells were treated with HDS for 24 h. Then the intracellular RNR was extracted and measured by LC–MS/MS system. **F** MM cells were treated with HDS (0, 50, and 100 µM) for 24 h. Then the intracellular dNTPs were extracted and measured. Data are presented as the means ± SD of 3 independent experiments. **P < 0.01, ***P < 0.001

Further experiments were carried out to confirm whether HDS targeted RRM2. We measured the interaction between HDS and RRM2 using NMR. Ligand-observed T1ρ NMR indicated that HDS interacted with RRM2, with notable signal broadening of HDS protons following the addition of RRM2 protein (Fig. 2A). This result was further confirmed by STD spectrum data (Fig. 2B). We also explored the specific interaction between HDS and RRM2 by SPR experiments, which showed a strong affinity with a binding affinity value of 27.5 µM between HDS and RRM2 (Fig. 2C), but not RRM1.

We further investigated the binding activity of HDS and RRM2 or RRM1 at the cellular level by CETSA. The thermal stability of RRM1 was increased in the presence of gemcitabine, which was known to bind to RRM1 (Fig. 2D), while the thermal stability of RRM2 was increased by HU known to bind to RRM2 (Fig. 2D). PCNA protein was a loading control which bound to DNA and retained thermal stability. In contrast, HDS treatment did not affect the thermal stability of RRM1 or PCNA (Fig. 2D). However, HDS increased the thermal stability of RRM2 (Fig. 2D), suggesting a specific physical interaction between HDS and RRM2.

We, therefore, examined RNR inhibition by HDS in MM cells. RNR activity was effectively suppressed in both MM cell lines following HDS treatment in a dose-dependent manner (Fig. 2E). We also examined levels of dNTPs, which are catalyzed by RNR, in MM cells treated with HDS. HDS significantly reduced dNTP levels in both H929 and ARP-1 cells (Fig. 2F). Overall, these data indicated that the intracellular dNTP pool in MM cells was perturbed by HDS, supporting the idea that HDS inhibited RNR activity in MM cells.

### RNR played an oncogenic role in MM cells

**Fig. 3:**
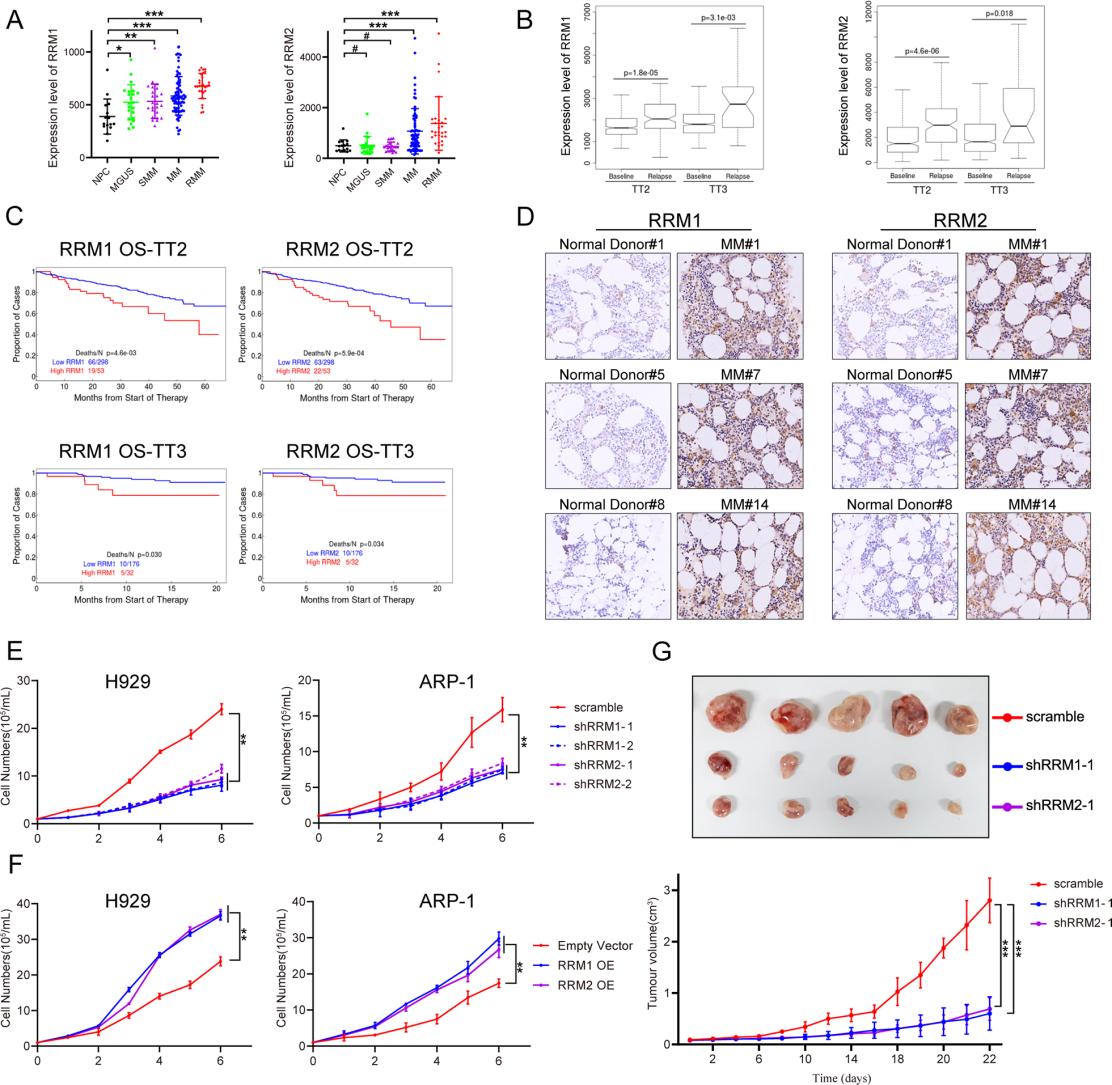
The oncogenic roles of RRM1 and RRM2 in MM. ** A** Analysis of RRM1 and RRM2 expression in publicly available MM patient data sets from Mayo Clinic (data set GSE6477). Increased RRM1 or RRM2 expression is observed in plasma cells from patients with MGUS, SMM, MM and relapsed MM than from normal healthy donors. **B** The expression levels of RRM1 and RRM2 were significantly up-regulated in relapsed patients from TT2 and TT3 cohorts in comparison with patients at the baseline stage (based on data set GSE2658). **C** Kaplan-Meier analyses of OS about patients from TT2 (p < 0.001) and TT3 (p < 0.05) cohorts revealed inferior outcomes among the patients with high (quartiles 4) RRM1 or RRM2 expression compared with the remaining patients with low (quartiles 1–3) RRM1 or RRM2 expression (based on data set GSE2658). **D** Immunohistochemical analysis of RRM1 and RRM2 expression (positive cells are brown) in 3 representative BM specimens derived from normal and MM patients (ND#1, 5, 8 and MM#1, 7, 14). Original magnification ×20. **E** MM cell lines were cultured for 6 days and knockdown of RRM1 or RRM2 in MM cell lines induced significant growth inhibition. **F** MM cell lines were cultured for 6 days and overexpression of RRM1 or RRM2 in MM cell lines induced significant growth increase. **G** Differences in tumor size between different groups of nude mice on Day 22 after injection of H929 cells. H929 cells transduced with RRM1 or RRM2 shRNA and scramble control vectors were subcutaneously injected into mice (n = 5/group). Tumor volume was quantified and knockdown of RRM1 or RRM2 inhibited tumor growth. All data are expressed as means ± SD of three independent experiments. *P < 0.05, **P < 0.01, ***P < 0.001

To further characterize RNR (RRM1 and RRM2) expression in MM, we evaluated public gene expression profiling (GEP) datasets and found that the expression of RRM1 and RRM2 was higher in MM patients compared with healthy donors (Fig. 3A). We also evaluated RRM1 and RRM2 expression levels in 51 paired MM samples obtained at baseline and relapse using GEP in total therapy 2 (TT2) and total therapy 3 (TT3). RRM1 and RRM2 were both significantly increased in relapsed MM samples compared with samples collected at diagnosis (Fig. 3B). In addition, RRM1 and RRM2 expression levels were higher in newly diagnosed patients with genetically defined high-risk compared with low-risk MM (Additional file 1: Fig. S2A). Higher expression levels of RRM1 and RRM2 were associated with significantly shorter overall survival in both TT2 and TT3 trials (Fig. 3C). We further investigated the biological relevance of RNR (RRM1 and RRM2) in MM by immunohistochemical staining of BM biopsies from 20 MM patients and 10 healthy donors. Both RRM1 and RRM2 showed higher expression levels in BM tissues from MM patients compared with healthy donors (Fig. 3D). We also examined the protein expression of RRM2 in multiple myeloma cells by western blotting. Our results showed that RRM2 protein expression was significantly higher in all MM cell lines (including H929, OPM2, ARP-1, RPMI 8226, MM.1 S, OCI-MY5) than in normal PBMCs (Additional file 1: Fig. S2B).

To define their biological role(s) in MM cells, we found RRM1 or RRM2 knockdown significantly inhibited MM cell growth (Fig. 3E) and increased apoptotic cell death (Additional file 1: Fig. S2C). Conversely, RRM1 or RRM2 overexpression increased cell proliferation (Fig. 3F). We also investigated the functions of RRM1 or RRM2 in vivo and found that tumor growth was inhibited following knockdown of RRM1 and RRM2, compared with the control group (Fig. 3G). These findings indicated that RNR activity was increased in MM. The RRM1/2 subunits were both overexpressed in MM and functioned as oncogenes conferring disease progression and aggressiveness, and may thus provide effective therapeutic targets. HDS inhibition of RNR activity by targeting RRM2 could thus be an effective therapeutic approach for MM.

### HDS impaired DNA damage repair by inhibiting dNTP synthesis

**Fig. 4:**
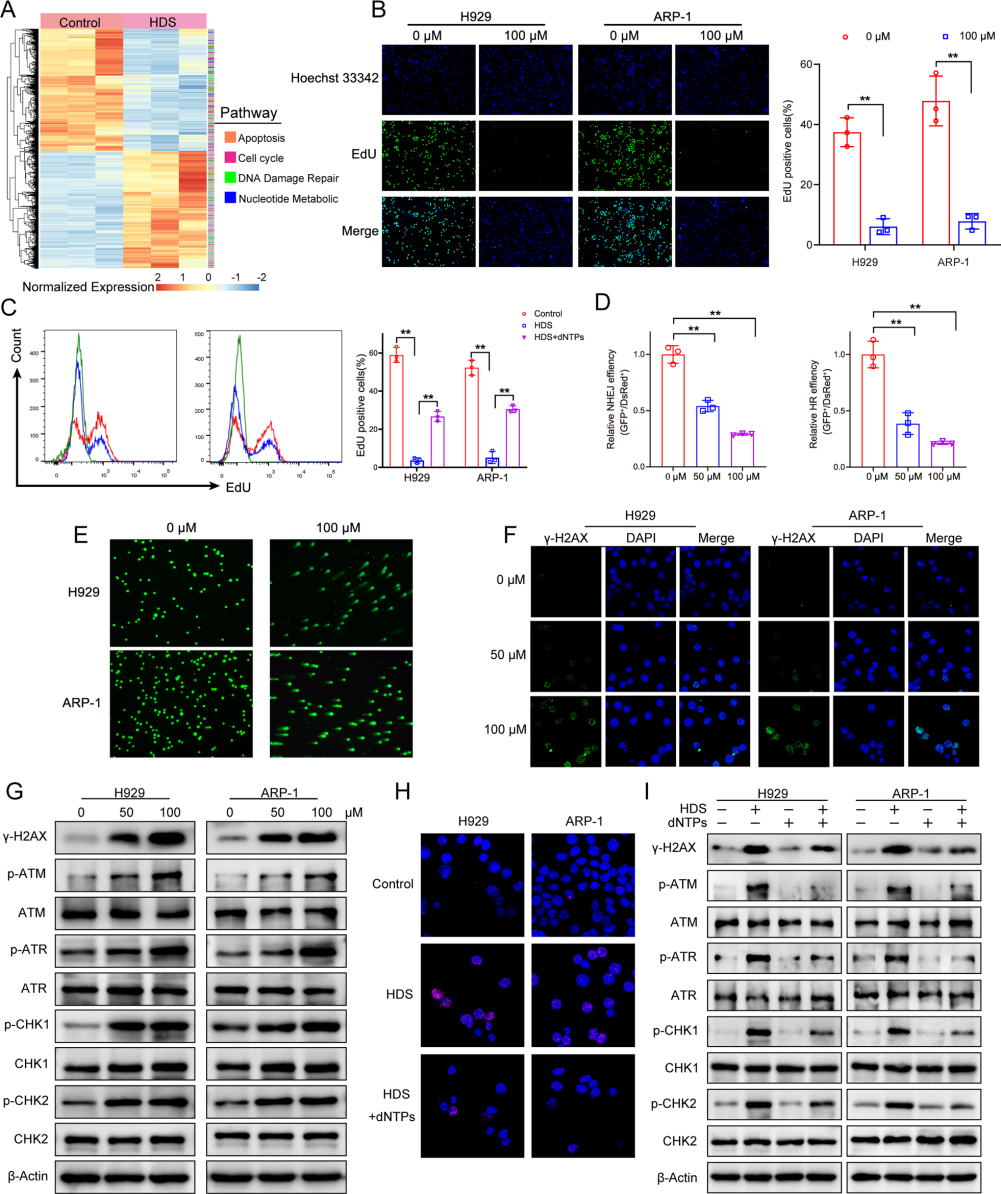
HDS impaired DNA damage repair by inhibiting dNTP synthesis. **A** Gene expression profiling was performed after H929 cells were treated with 50 µM HDS for 48 h. Heat map showed significant pathways affected by HDS. **B** DNA synthesis in MM cells treated with HDS for 24 h was evaluated by EdU incorporation. EdU incorporation was observed using laser scanning confocal microscopy. The number of EdU-positive cells was quantified. **C** MM cells were incubated with HDS either without or with exogenous 50 µM dNTPs for 24 h. Percentage of EdU positive cells in flow cytometry was showed in the right panel. **D** NHEJ and HR in cells treated with HDS (0, 50 and 100 µM) for 24 h were quantified by the GFP and DsRed expression using flow cytometry. **E** MM cells were treated with 100 µM HDS for 24 h, and then the comet assay was performed. **F** Expression of cellular γ-H2AX in H929 and ARP-1 cells treated with or without 100 µM HDS for 24 h was detected by immunofluorescence. **G** Western blot analysis of DNA damage-related proteins in cell lysates of H929 and ARP-1 cells treated with indicated concentration of HDS for 24 h. **H** Immunofluorescence staining of cellular γ-H2AX in H929 and ARP-1cells treated with 100 µM HDS, either without or with 50 µM exogenous dNTPs for 24 h. **I** Western blot analysis of DNA damage-related proteins in MM cells incubated with HDS either without or with exogenous 50 µM dNTPs for 24 h. All data are expressed as means ± SD of three independent experiments. *P < 0.05, **P < 0.01, ***P < 0.001

RNR is the only enzyme that catalyzes the conversion of dNTPs, which are essential for DNA synthesis and DNA repair [27]. We performed a global gene expression profiling analysis of H929 cells after incubation with 50 µM HDS for 48 h. Gene-set enrichment analyses (GSEAs) identified dysregulated DNA damage repair and nucleotide metabolism (Fig. 4A). We examined the kinetics of dNTP incorporation during DNA replication by 5-ethynyl-2′-deoxyuridine (EdU) incorporation into DNA. HDS decreased the EdU-positive cell population, suggesting a low DNA synthesis efficiency (Fig. 4B; Additional file 1: Fig. S3A). Notably, the HDS-induced impairment of DNA synthesis was partially restored by dNTP supplementation (Fig. 4C). We next examined DNA repair using a GFP-based reporter assay to measure the efficiency of Non-Homology End Joining (NHEJ) and Homologous Recombination (HR) repair [27, 28]. Consistent with GSEAs, HDS treatment induced HR and NHEJ deficiency in a concentration-dependent manner (Fig. 4D). HDS thus resulted in a global decrease in available cellular dNTPs, leading to dual inhibition of HR and NHEJ.

Based on the above observation, we considered that the inhibition of HR and NHEJ by HDS could result in the accumulation of unrepaired DSBs. Obvious comet tails in comet assay indicating a significant number of DSBs were observed in HDS-treated MM cells (Fig. 4E). During the response of mammalian cells to DSBs, γ-H2AX occurs in foci at sites proximal to the DNA breaks [29]. In this study, γ-H2AX levels were increased in MM cells treated with HDS compared with baseline levels, suggesting ongoing DNA damage (Fig. 4F). Immunoblots also showed that HDS triggered DNA damage in MM cells, including the increase of γ-H2AX, phosphorylated (p)-ATM, and p-ATR, as well as their downstream effectors p-Chk1 and p-Chk2 (Fig. 4G; Additional file 1: Fig. S3B). However, both tested cell lines showed substantial decreases in γ-H2AX foci following exogenous dNTP supplementation (Fig. 4H). In addition, exogenous dNTPs also relieved HDS-induced apoptosis (Additional file 1: Fig. S4) and DNA damage response protein expression (Fig. 4I).

### Combined treatment with HDS and melphalan or bortezomib induced synergistic anti-myeloma activity

**Fig. 5:**
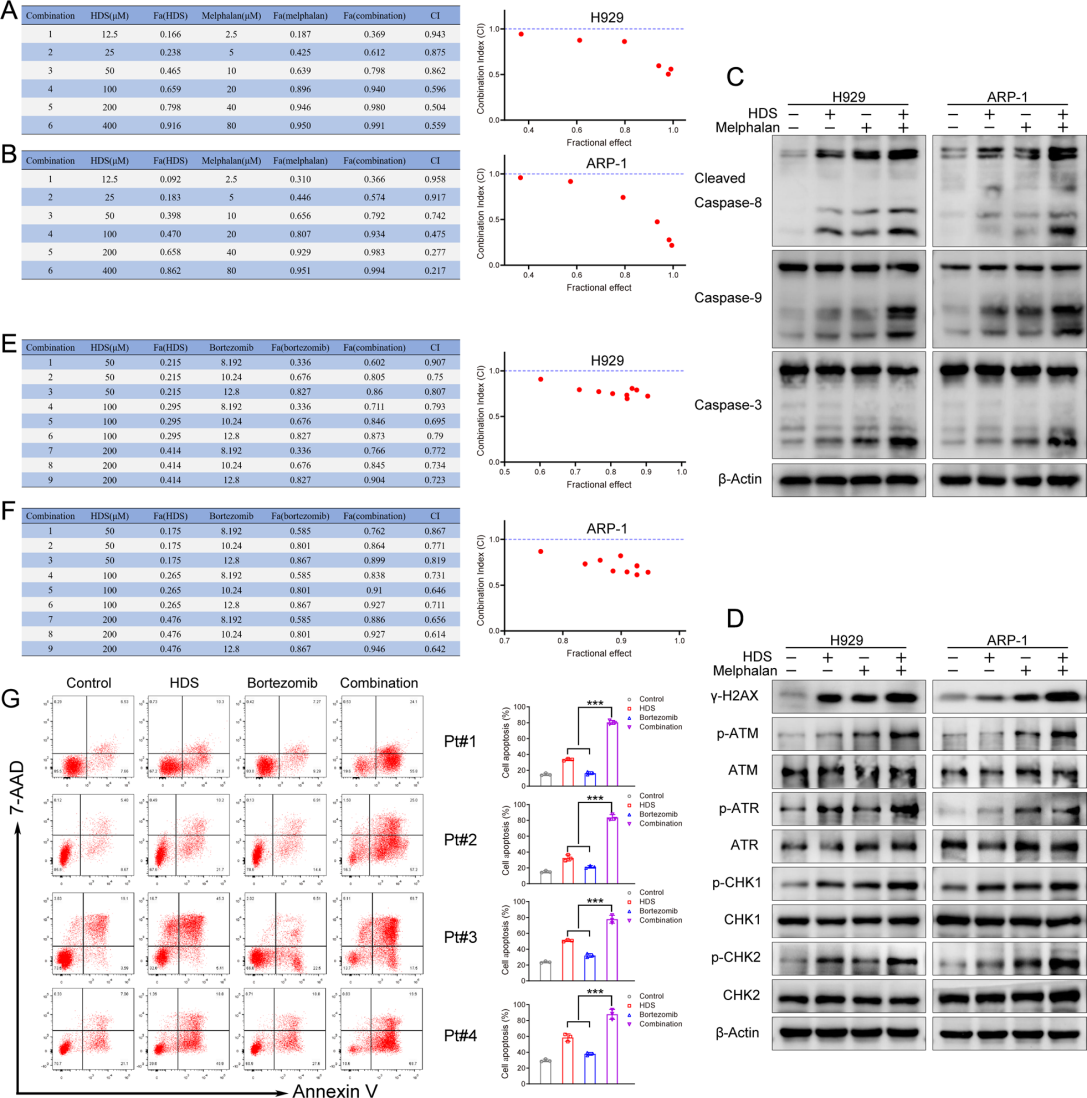
Synergistic effect of HDS with melphalan and bortezomib. ** A** H929 and **(B)** ARP-1 cells were co-treated with indicated concentration of HDS (12.5–400 µM) and various concentrations of Melphalan (2.5–80 µM) either alone or in combination for 48 h. Cell viability was assessed using a CCK-8 assay (left panel). CI values were calculated based on the median-effect principle. The right graph showed values from the left tables. CI < 1 indicated synergism of HDS and melphalan, as determined using CalcuSyn software. **C**,** D** The expression of relative apoptosis (**C**) and DNA damage proteins (**D**) of MM cells were monitored by western blot after treated with HDS (50 µM) in the presence (+) or absence (−) of melphalan (10 µM) alone or together. Representative results of triplicate experiments are shown. **E**, **F** H929 (**E**) and ARP-1 (**F**) cells were treated with bortezomib for 24 h, and then HDS was added for an additional 24 h. Cell viability was assessed using a CCK-8 assay (left panel). CI values were using CalcuSyn software (Right panel). **G** Primary CD138^+^ cells were isolated from four bortezomib-refractory patients. MM cells were treated with HDS (40 µM) and bortezomib (40 nM) alone or in combination for 48 h, followed by an assessment of cell apoptosis. Representative results of triplicate experiments were shown (left panel). Apoptotic cells were quantified on the right. Data are expressed as means ± SD of three independent experiments. ***P < 0.001

As a DNA-damaging agent, melphalan is widely used as a preparative agent in patients with MM undergoing autologous stem cell transplantation [30]. The DNA repair capacity of MM cells represents an important mechanism of resistance to melphalan [31, 32]. Given that HDS directly impaired HR and NHEJ mediated DSB repair, we considered the possible effects of combined treatment with HDS and melphalan. Melphalan-induced cytotoxicity was enhanced by increasing concentrations of HDS (Fig. 5A, B), and combination index (CI) values calculated using CalcuSyn software suggested that the combination of HDS with melphalan induced synergistic cytotoxicity in MM cells (CI < 1) [33]. Consistent with cytotoxicity, HDS with melphalan also markedly upregulated the cleavage of caspase-3, -8, and − 9, suggesting that the combined treatment enhanced apoptosis (Fig. 5C). The combination treatment also activated DNA damage more strongly than monotherapy (Fig. 5D). Furthermore, the combination of bortezomib and HDS also demonstrated synergistic anti-myeloma activity, including inducing apoptosis by upregulating cleavage of caspase-3, -8, and -9 (Fig. 5E, F; Additional file 1: Fig. S5). For further validation, we also examined the effect of combined treatment in primary CD138^+^ cells from bortezomib-refractory patients. High-dose bortezomib slightly induced MM cell apoptosis, whereas simultaneous treatment with HDS and bortezomib resulted in markedly higher apoptosis levels (Fig. 5G).

#### HDS inhibited myeloma growth in vivo

**Fig. 6:**
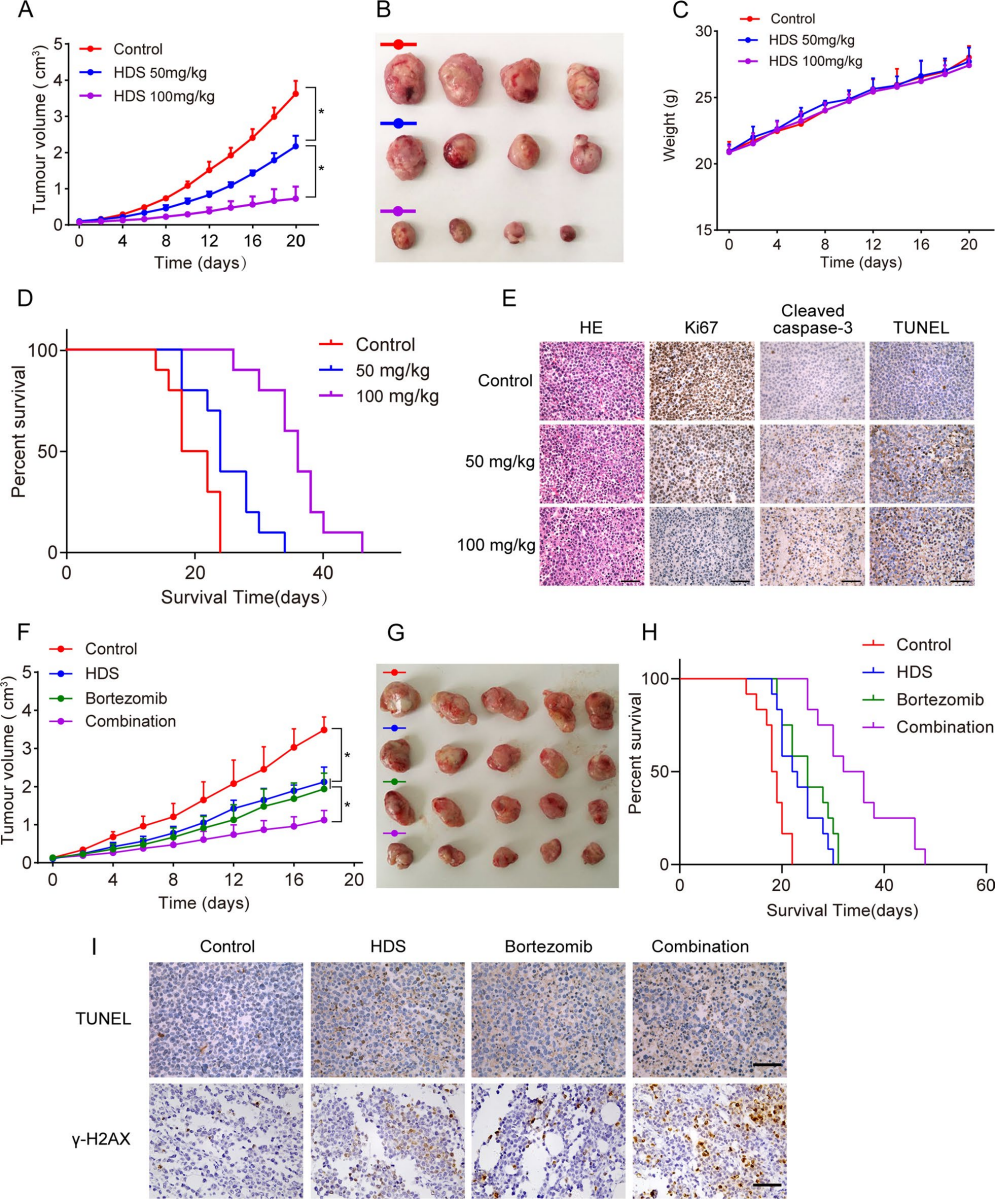
HDS inhibited myeloma growth ***in vivo***.** A** Nude mice bearing H929 tumors were daily given either HDS (50 mg/kg, 100 mg/kg) or vehicle through intravenous injection for 20 days. **B** Tumors on day 20. **C** The weight of mice. **D** Kaplan-Meier analyses of overall survival. **(E)** Tumor sections were stained with HE, Ki67, cleaved caspase-3 or TUNEL. The positive cells in tumor sections stained with Ki67, cleaved caspase-3 or TUNEL are the dark brown ones. Scale bars, 100 µM. **F** Nude mice bearing H929 tumors were intravenously injected with vehicle (daily), HDS (50 mg/kg, daily), bortezomib (0.5 mg/kg, every three days) or HDS (50 mg/kg, daily) plus bortezomib (0.5 mg/kg, every 3 days). Tumor volumes were showed. **G** Pictures of tumors in nude mice. **H** Using Kaplan-Meier and log-rank analysis, the median overall survival of animals treated with combination therapy was significantly prolonged. **I** Tumor sections were stained with TUNEL and γ-H2AX. Positive cells are the dark brown ones. Scale bar, 100 µM. Data are represented as means ± SD. *P < 0.05

We administered HDS or vehicles daily to mice by intravenous injection. Tumor growth was suppressed in HDS-treated mice even at the dose of 50 mg/kg (Fig. 6A, B), suggesting the anti-MM activity of HDS in vivo. No significant weight change was observed in drug-treated animals, indicating that HDS was well tolerated (Fig. 6C). HDS also significantly prolonged overall survival compared with vehicle-treated animals (Fig. 6D). Hematoxylin and eosin (H&E) staining showed that HDS induced cell shrinkage and fragmentation in tumors. Moreover, indicated by Ki67 staining, tumor proliferation was reduced. Apoptosis was increased as suggested by the increase of cleaved caspase-3 and TUNEL-positive cells (Fig. 6E). There were no significant histologic changes in the heart, liver, spleen, lungs, and kidneys of HDS treated mice, as detected by H&E staining, indicating that HDS had minimal side effects (Additional file 1: Fig. S6).

We further investigated the combined effects of HDS and bortezomib in vivo. Combined treatment induced more effective tumor inhibition compared with treatment with HDS or bortezomib alone (Fig. 6F, G). Compared with monotherapy, combined treatment also significantly prolonged survival in mice (Fig. 6H). TUNEL and γ-H2AX assays showed more positive cells following combined treatment of HDS with bortezomib, further supporting our observations (Fig. 6I).

### HDS exhibited anti-tumor activity in MM patients

**Fig. 7:**
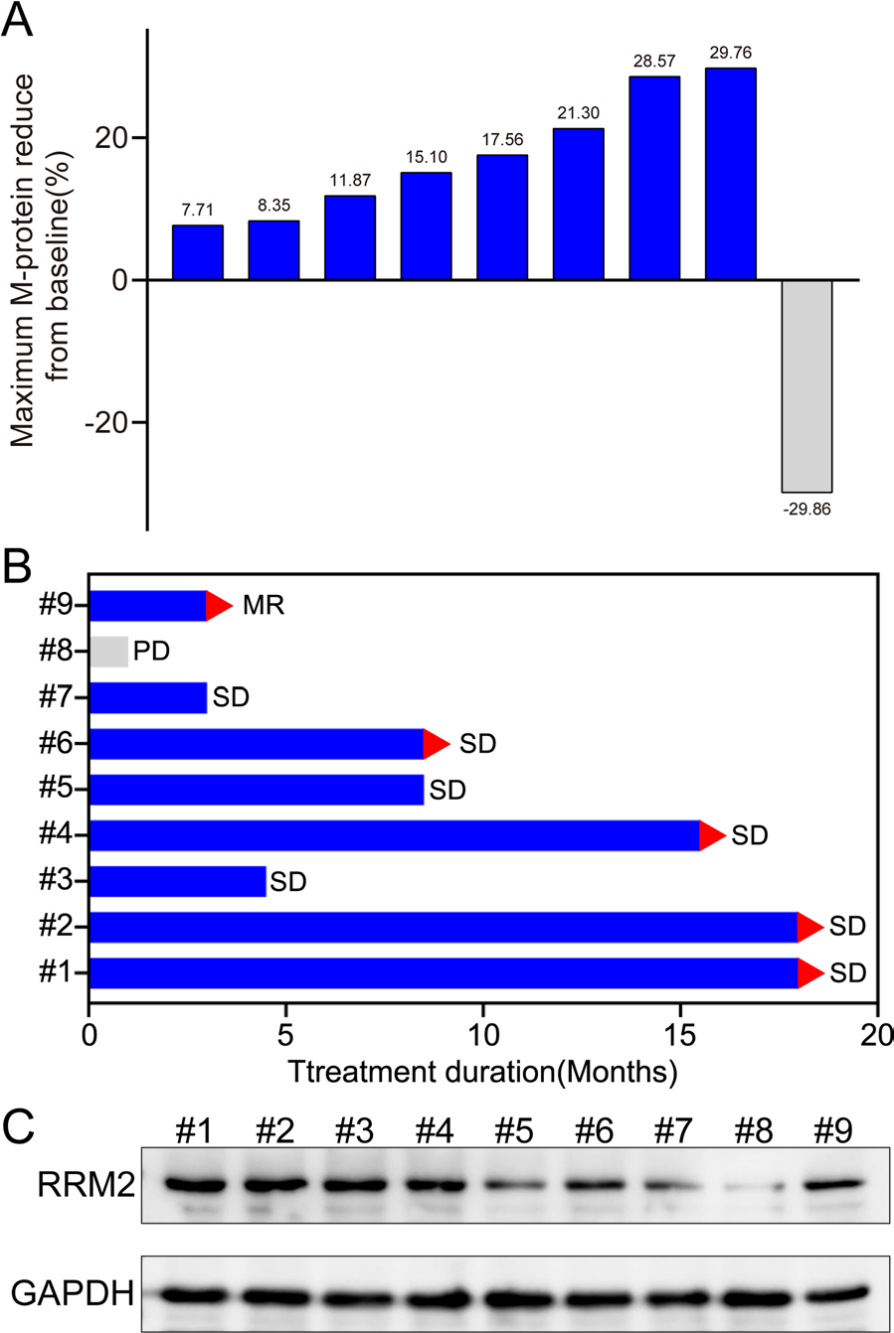
Clinical activity of HDS in MM patients. **A** The maximum change of M-protein from baseline level after HDS treatment. **B** Swim‐lane plot showed the treatment response and duration for 9 MM patients after HDS treatment. Arrows indicated patients who were still ongoing at the time of study closure. *PD* progressive disease, *SD* stable disease, *MR* minimal response. **C** The protein levels of RRM2 in CD138^+^ MM cells obtained from patients were evaluated, with GAPDH used as a loading control

We evaluated the safety and clinical activity of HDS in MM patients in an open-label phase I clinical trial (ClinicalTrials.gov: NCT03670173). Nine MM patients were enrolled and the patient characteristics were shown in table S2. All patients were included in the evaluation of adverse effects, irrespective of the therapy duration. HDS was well tolerated, with no serious treatment-related adverse events in any MM patients. No dose-limiting toxicity was observed during the study, and the maximum tolerated dose was not reached. Before initiation of treatment, patients #3, #7, #9 had grade 3 anemia and patients #1, #2, #8 had grade 2 anemia at baseline. Patient #2 with grade 2 anemia recovered to grade 1 after HDS treatment. No patient had an increase in anemia grade. As for side effects, patient #3 had grade 2 creatinine elevation which led to discontinuation of HDS administration. The most common nonhematological adverse effects were fatigue and nausea. Patients #2, #9 had grade 1–2 rapidly reversible nausea, but no HDS-related grade 3 or 4 adverse events were observed. There were no other clinically meaningful treatment-related changes in hematologic and vital signs, body weight, and electrocardiogram results. The maximum change in the level of M-protein after treatment from baseline was shown in Fig. 7A. Patient #8 failed to respond to HDS and had progressive disease during the first cycle of HDS. Patient #9 had a minimal response (MR), classified according to the International Myeloma Working Group criteria based on *post hoc* analysis of changes in M-protein levels. The other MM patients had prolonged stabilization of their disease, with three (33.3%) patients achieving stable disease states for > 500 days (Fig. 7B). BM aspirates were obtained from patients before HDS administration and MM cells were isolated using anti-CD138-coated microbeads for immunoblotting. Notably, patient #8 who showed the lowest expression level of RRM2 protein, did not respond to HDS (Fig. 7C).

Although the number of patients treated in this study was small, single-agent HDS demonstrated evidence of disease stabilization. The absence of myelosuppression and other important treatment-related adverse effects, as well as the clinical activity profile demonstrated in the present trial, indicate that the combination of HDS with various standard agents might be feasible and warrant clinical study. Larger studies of HDS, its analogues, and other inhibitors of RNR are therefore warranted in patients with myeloma.

## Discussion

MM remains a largely incurable disease with drug resistance, making it urgent to explore new drugs with good efficacy [34]. Unfortunately, developing a new drug is expensive, time-consuming, and often ends in failure [35]. Repurposing approved drugs with established toxicity and safety profiles in animals and humans could significantly decline the expense and time of drug development. HDS has been used to promote bile drainage and protect liver function in clinical practice for decades [19, 36]. In this study, we evaluated for the first time the anti-multiple myeloma potential of HDS in models *in vitro* and in vivo, as well as in clinical patients. We found that HDS displayed efficient antimyeloma activity in a large panel of MM cells, including primary myeloma cells from patients. Our research provides a new idea for the clinical application of HDS and a new strategy for the treatment of MM patients.

Our results showed that HDS treatment resulted in inhibition of cell viability in all tested MM cell lines, suggesting the generalized antitumor effects of HDS on MM cells. Therefore, we made a further in-depth investigation into the anti-MM mechanism of HDS. High activity of RNR is necessary to supply the dNTPs required for the rapid proliferation of cancers, and increased RNR activity is accordingly frequently observed in cancer [7]. Our results of the LC–MS/MS system demonstrated that HDS significantly inhibited the activity of RNR in MM cells. Interestingly, the minimally catalytically active form of RNR comprises two pairs of RRM1 and RRM2 subunits, respectively [10]. Based on the results of NMR and SPR, we found HDS bound with high affinity to RRM2 and interacted with RRM2. In contrast, HDS did not exhibit obvious binding capability with RRM1, consistent with the CETSA results at the cellular level. Our results showed that knockdown and overexpression of RRM2 affected cytotoxic effects of HDS in MM cells, whereas RRM1 protein levels had no such effect on HDS activity, thus demonstrating the specificity of HDS for RRM2.

It is well known that dNTP production is critical for cell survival, and cancer cells depend more on an adequate supply of dNTPs than normal cells to maintain their malignant proliferation [16, 17]. As RNR is the rate-limiting enzyme catalyzing the formation of dNTPs from NTPs, its inhibition by HDS would affect dNTP synthesis. Our Results showed that dNTP levels were significantly reduced in HDS-treated MM cells. Furthermore, dNTPs are not only required for DNA synthesis but also for DNA repair [37]. MM cells with persistent DNA damage are dependent on DNA repair pathways [38, 39], thus making MM cells more sensitive to dNTP production [10]. The results of our in vitro experiments showed that HDS significantly reduced dNTP levels in H929 and ARP-1 cells, and excessive exogenous supplementation of dNTPs can partially inhibit the anti-tumor effect of HDS, suggesting that HDS exerted an anti-MM effect via the inhibition of RNR activity through targeting its subunit RRM2 and consequently depletion pool of dNTPs. Constitutive ongoing intrinsic DNA damage occurs in hematologic malignancies, including MM [39], and the DNA replication pathway was significantly enriched in MM cells with enhanced DNA damage [40]. Dysregulated DNA repair can lead to survival advantage and drug resistance among cells, with increased mutation rates and progressive accumulation of genetic variation over time [41, 42]. The RRM2 subunit of RNR localizes to the nucleus in response to DNA damage to ensure local dNTP production for efficient DNA repair synthesis [8, 43, 44]. Intracellular levels of dNTPs are tightly controlled and increase 20-fold during the replicative S phase and after DNA damage [45]. The subsequently increased dNTPs following RNR activation often result in reduced fidelity of DNA replication, leading to increased gene mutation rates and genome instability [10]. Our results showed that HDS inhibited cellular RNR activity by targeting RRM2 and impaired dNTPs synthesis. Low dNTP pools caused by HDS further led to reduced efficiency of NHEJ and HR repair pathways. We hypothesized that dual inhibition of NHEJ and HR by HDS might result in the accumulation of unrepaired DSBs in MM cells. Indeed, our results showed that HDS treatment resulted in huge DNA damage accumulation as suggested by increased γ-H2AX protein levels and foci. The accumulation of unrepaired DNA damage will, in turn, initiate programmed cell death [46]. Furthermore, this increased DNA damage was partially abrogated by excessive exogenous dNTP supplementation. These data further indicated that dNTP synthesis impairment was an essential mechanism responsible for the effect of HDS.

P53 abnormality is an independent poor prognostic factor, which is associated with disease progression and drug resistance in MM [47]. Our results showed that HDS displayed growth inhibition in all tested cell lines, regardless of p53 status. On one hand, the accumulated DNA damage caused by HDS can promote cell apoptosis by activating p53 [22]; on the other hand, the accumulated DNA damage can promote cell death in a p53-independent pathway. For example, although p53 plays a central role in DNA damage-induced cell death, p53-mutant cells may still die from DNA damage due to catastrophic mitotic death [48, 49]. Moreover, it has been reported that after DNA damage, ABL1 kinase complexed with YAP1 in the nucleus can also induce apoptosis in the pathway independent of p53 [50]. Although the specific molecular mechanism for cell death after HDS-induced accumulation of DNA damage in MM cells is not clear, our results show that HDS can induce apoptosis in p53-wild and mutant MM cells. Therefore, HDS may be clinically applicable to patients with p53 mutations, but this requires further exploration and validation.

Interestingly, we demonstrated a significant difference in drug effects between malignant and normal cells. HDS showed no significant cytotoxic effect on normal PBMCs and was well tolerated in vivo. In our in vitro/vivo studies, we used the dosage of 50–100 µM HDS, which was a relatively high concentration. As the results of our cellular experiments showed, this concentration exhibited low toxicity in normal cells, making it possible for HDS to be applied in clinical patients. Indeed, as an old clinical drug, no serious adverse events about HDS have been reported in clinical use for hepatobiliary diseases, as well as in our clinical trial.

In our clinical trial, patient #8 had a low level of RRM2 and did not respond to HDS. Based on our cellular and molecular results, HDS mainly bound to RRM2 and inhibited NHEJ and HR repair in MM cells by reducing the production of dNTPs, resulting in the accumulation of DNA damage in MM cells, thus inhibiting MM cell proliferation and inducing apoptosis. Therefore, patient #8 who showed a low expression level of RRM2 did not respond to HDS may be due to a lack of binding target, further suggesting that HDS played an anti-tumor effect mainly through targeting RRM2.

Tumor cells develop chemoresistance via DNA repair pathways, and the ability of MM cells to remove melphalan adducts through DNA repair pathways represents an important mechanism of resistance to melphalan therapy [32]. Notably, responders to melphalan therapy are characterized by slower rates of DSB repair [31, 32]. A connection between chemotherapy resistance and enhanced DNA repair has also been documented in other malignancies [51]. The translational significance of HDS is further underlined by its synergism with the commonly used agents in MM treatment. Exposure to DNA damaging agents such as melphalan whilst simultaneously targeting their DNA damage repair mechanisms may thus represent an attractive combinational strategy. The present results indicated that the combination of HDS with melphalan induced synergistic cytotoxicity in MM cells. We further confirmed the synergistic effects of HDS and bortezomib in vitro and in vivo. Bortezomib treatment has been reported to induce “BRCAness” in MM and impair the HR pathway, with no significant effect on NHEJ [52]. Dual inhibition of NHEJ and HR by HDS, therefore, potentiated the efficacy of bortezomib. These data thus provide a framework for the combined use of HDS with novel and conventional anti-MM agents in clinical practice.

## Conclusions

In conclusion, HDS displays antitumor activity in MM, via the inhibition of RNR activity through targeting RRM2. The efficacy and low toxicity demonstrated in the current studies provide a rationale for further clinical evaluations of HDS as an anti-MM agent, and the possible repurposing of this traditional drug for new therapeutic interventions. In addition, HDS is an inexpensive and readily available drug that may be particularly attractive for patients in resource-poor countries in which standard-of-care myeloma drugs are unavailable or out of reach for many patients.

## Supplementary Information

**Additional file 1.**Supplementary Materials and Figures.

## Data Availability

The datasets supporting the conclusions of this article are included in this published article (and its Additional files).

## Abbreviations

MM: Multiple myeloma
RNR: Ribonucleotide reductase
dNTPs: Deoxyribonucleotides
HDS: 4-Hydroxysalicylanilide
RRM2: RNR subunit M2
DSBs: DNA double-strand breaks
BM: Bone marrow
dNDPs: Deoxyribonucleoside diphosphates
HU: Hydroxyurea
NMR: Nuclear magnetic resonance
STD: S aturation transfer difference
SPR: Surface plasmon resonance
CETSA: Cellular thermal shift assay
LC–MS/MS: Liquid chromatography/tandem mass spectrometry
NHEJ: Non-homology end joining
HR: Homologous recombination
PD: Progression of disease
PBMCs: Peripheral blood mononuclear cells
BMSCs: BM stromal cells
TUNEL: Terminal deoxynucleotidyl transferase dUTP nick and labeling
GEP: Gene expression profiling
GSEAs: Gene-set enrichment analyses
EdU: 5-Ethynyl- 2′-deoxyuridine
CI: Combination index
