# Supplementary material for: Preclinical validation and phase I trial of 4-hydroxysalicylanilide, targeting ribonucleotide reductase mediated dNTP synthesis in multiple myeloma
Source: J Biomed Sci. 2022 May 12;29:32. doi: 10.1186/s12929-022-00813-2 (PMC9097096; doi:10.1186/s12929-022-00813-2)
Supplement: Supplementary file 1 — Additional file 1.Supplementary Materials and Figures. [file 12929_2022_813_MOESM1_ESM.docx]

**Supplementary Materials and Methods**

**Cell lines**

The MM cell lines ARP-1, OPM2, OCI-My5, MM.1S, U266, the bortezomib-sensitive MM cell lines H929 and RPMI 8226, the bortezomib-resistant MM cell lines H929R and RPMI 8226/R5, and HS15 were kindly provided by researchers or purchased from American Type Culture Collection (ATCC) (Manassas, VA, USA). Primary cells were acquired from bone marrow (BM) samples of MM patients and the peripheral blood of healthy donors. Subsequently, BM samples and peripheral blood were subjected to Ficoll-Hypaque density gradient centrifugation, and the bone marrow mononuclear cells and PBMCs were obtained. CD138^+^ MM cells were then isolated from bone marrow mononuclear cells using human CD138 microbead (Miltenyi Biotech, Auburn, CA). Informed consent was obtained from each patient and healthy donor. These studies have been approved by the Review Board and Ethics Committee of Shanghai Tenth People’s Hospital. Cells were cultured in RPMI-1640 medium (Gibco, Carlsbad, CA, USA) containing 100 units/mL penicillin, 100 μg/mL streptomycin and 10% fetal bovine serum (Gibco, Carlsbad, CA, USA). 293T cell line was purchased from ATCC and cultured in DMEM medium (Gibco, Carlsbad, CA, USA) containing 100 units/mL penicillin, 100 μg/mL streptomycin and 10% fetal bovine serum (Gibco, Carlsbad, CA, USA).

**Cell Viability Measurement**

MM cells were seeded into 96-well plates (2 × 10^5^ /100 μL), and treated with drugs at different concentrations for the indicated time. Cell viability was determined using a CCK8 assay (Cell Counting Kit-8, Dojindo, Kumamoto, Japan) following the manufacturer’s protocol.

**Apoptosis Detection**

Cells were incubated in 24-well plates and exposed to different concentrations of HDS for the indicated time. Subsequently, cells were stained with Annexin V/ propidium iodide (PI) (BD Pharmingen, Franklin Lakes, USA). The staining was performed according to the manufacturer’s instructions. After staining, cell apoptosis was analyzed using a BD FACSCanto II flow cytometer (BD BioScience, San Jose, CA, USA).

**Cell Cycle Analysis**

After treatment with different concentrations of HDS for the indicated time, MM cells were harvested, washed with cold PBS, permeabilized using 70% ethanol overnight at −20 °C, and then incubated with 300 μL PI/RNase staining buffer (BD Pharmingen, Franklin Lakes, NJ, USA) at room temperature for 15 min followed by flow cytometric analysis. Data was analyzed using ModFit LT software.

**Western Blotting**

Cells were collected, washed with cold PBS, and lysed with lysis buffer (100 mM Tris-HCl, pH 6.8, 4% SDS, 20% glycerol) on ice for 30 min. Protein concentrations were determined using Pierce™ BCA Protein Assay Kit (Thermo fisher Scientific, #23227). Primary antibodies including cleaved caspase-8, caspase-9, caspase-3, β-actin, and γ-H2A.X were from Cell Signaling Technology (CST, Beverly, MA, USA). RRM1, RRM2, PCNA, ATM, phospho-ATM (S1981), ATR, phospho-ATR (S248), CHK1, phospho-CHK2, were from Abcam (Cambridge, UK).

**Immunohistochemistry**

The paraffin-embedded tumor tissue sections were dewaxed and rehydrated for IHC, and the antigen was retrieved by heating in an autoclave in 0.01 M sodium citrate buffer (pH 6.0). After pre-incubation with normal goat serum for 40 min at 37 °C to block nonspecific binding sites, the slides were incubated with primary antibodies at 4 °C overnight. The next day, slides were incubated with secondary antibodies at room temperature for 1 h, then visualized using diaminobenzidine peroxide solution and counterstained with hematoxylin. Images of representative fields were taken by the microscope (Leica, Wetzlar, Germany). Primary antibodies RRM1, RRM2, and Ki67 were from Abcam (Cambridge, UK). Cleaved caspase-3 and γ-H2AX were from Cell Signaling Technology (CST, Beverly, MA, USA).

**5-ethynyl- 2′-deoxyuridine (EdU) Incorporation Assay**

MM cells were seeded in 24-well plates and treated with 100 μM HDS for 24 h. Then the EdU incorporation assay was performed following the manufacturer’s instruction using the EdU kit (RiboBio, Guangzhou, China). Briefly, cells were exposed to 50 μM EdU for 2 h at 37 ℃, washed with PBS and fixed with 4% paraformaldehyde, followed by permeabilization with 0.5% Triton-X. Subsequently, cells were incubated with azide-conjugated Alexa Fluor 567 dye and Hoechst 33342 for 30 min respectively and visualized under a laser scanning confocal microscopy, or cells were only stained with azide-conjugated Alexa Fluor 567 dye and analyzed by flow cytometry.

**DNA Repair Assays**

The efficiency of Non-Homology End Joining (NHEJ) or Homologous Recombination (HR) repair is measured using the GFP-based reporter systems. Plasmids containing the NHEJ or HR cassettes were linearized by I-Scel endonuclease and gel purified. Then the plasmids (linearized NHEJ/HR plasmids + DsRed plasmids) were transfected into H15C cells pretreated with HDS for 24 h using the electroporation kit (V4XP-2032) on a Lonza 4D machine (Cologne, Germany) (program DT-130). After incubation with HDS for another 48 h, cells were subjected to flow cytometer (BD BioScience) to analyze the expression of GFP and DsRed. GFP expression represents the occurrence of NHEJ or HR repair. DsRed expression was used as a control. The ratio of GFP/DsRed was calculated to represent the amount of NHEJ or HR repair occurring.

**Comet Assay**

MM cells treated with 100 μM HDS for 24 h were mixed with 0.5% low-melting-point agarose, applied to the slides and covered with 1% normal-melting agarose. After the low-melting-point agarose had solidified, the slides were submerged in pre-cooling neutral lysis buffer for 3 h at 4 °C. Subsequently, the slides were immersed in the pre-cooling neutral electrophoresis buffer for 20 min, followed by electrophoresis for 20 min under a voltage of 20 V/cm. Finally, the slides were stained with SYBR Green I and pictured under an inverted fluorescence microscope.

***In vivo* Animal Experiments**

BALB/C nude mice (5 weeks old) were purchased from Shanghai Laboratory Animal Center (Shanghai, China) and fed in the animal house of the Shanghai Tenth People’s Hospital. H929 cells (2 × 10^6^) in 100 μL serum-free culture medium were inoculated subcutaneously into the right flank of each mouse. When the tumors were measurable, mice were randomly divided into treatment and control groups. The mice in treatment groups were injected with HDS (daily) and/or bortezomib (every three days) via the tail vein. The control group received 200 μL vehicle consisting of 5% DMSO and 4% castor oil in saline. Tumor size and body weight were measured every other day. Tumor volume = 4π/3× (width/2)^2^ × (length/2). At the end of the treatment, mice were euthanized. All animal studies have been approved by the Review Board and Ethics Committee of Shanghai Tenth People’s Hospital (ID: SYXK 2011-0111).

**HDS clinical trial**

**Patients.** Key inclusion criteria were as follows: above 18 years old, diagnosed with active and measurable (symptomatic) MM according to IMWG 2003/WHO 2008(V4). MM diagnosis criteria were detailed as following: 1). Positive M protein in serum and/or urine; 2). Pathologically diagnosed with multiple myeloma or found colonic plasma cells in bone marrow; 3). At least one symptom of related organ damage or tissue lesion: a. hypercalcemia: serum calcium increases 0.25mmol/L or more over upper limit of normal value (ULN) or > 2.75mmol/L; b. anemia: Hemoglobin decreases 20 g/L or more over lower limit of normal value (LLN) or ＜100g/L; c. bone lesion: lytic bone lesion or osteoporosis accompanied with compressive fracture (confirmed with MRI、CT or PET-CT); d. others: symptomatic hyperviscosity, amyloidosis, recurrent infection (more than twice within 12 months); Eastern Cancer Organization Group (ECOG) score ≤ 2 and expected survival > 2 months; Belongs to “measurable disease”: serum M protein ≥10g/L and/or 24 hour urine M protein ≥ 200 mg; No severe organic dysfunction (except renal function insufficiency caused by multiple myeloma), lab results must meet the following criteria (within 7 days before initiation of therapy): a. Total bilirubin ≤ 1.5×ULN (same age group); b. AST and ALT ≤ 2.5×ULN (same age group); c. Cardiac enzyme < 2×ULN (same age group); d. Normal ejection fraction confirmed in echo;

Key exclusion criteria included received anti-myeloma treatment before (not include radiotherapy, bisphosphonates or single short term steroids treatment (the dose and duration of prednisone should be no more than 40 mg/d and 4 days and should discontinue this treatment within 14 days before the enrollment)); Primary or secondary plasma cell leukemia; Positive HIV tests or active infection phase of HAV, HBV and HCV; or HBV DNA copies ＞10^4^/ml; AST and ALT > 2.5×ULN (same age group); Severe diseases that threaten patients with unacceptable risks; Renal failure requiring hemodialysis or peritoneal dialysis; Severe embolic or thrombotic events before therapy; Major surgery within 30 days before being enrolled; Total obstruction of biliary tract; Glaucoma; History of malignancies except multiple myeloma unless being cured for more than 3 years; Severe allergic to HDS capsule; Gestation, lactation or disagreed pregnancy; Severe infectious diseases (uncured tuberculosis, pulmonary aspergillosis); Severe liver and kidney dysfunction; Patients who are considered unsuitable for enrollment by investigators.

Supplementary Figure S1


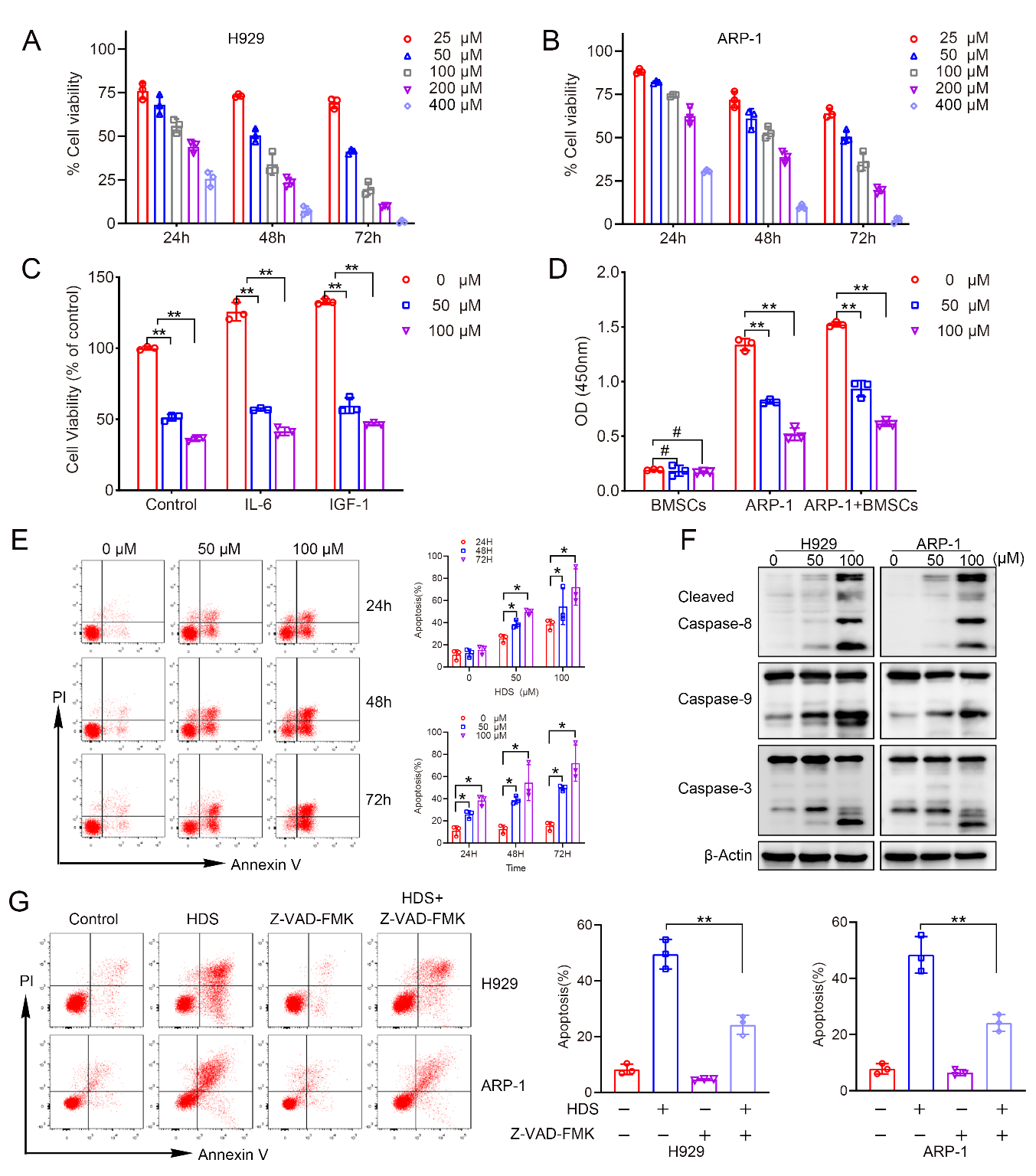


**Supplementary Fig.S1. Cytotoxic activity of HDS against MM cells.** **(A)** H929 and **(B)** ARP-1cells were treated with indicated HDS concentrations (25, 50,100, 200 and 400 μM) for 24, 48 and 72 h, and then analyzed for cell viability using CCK8 assay. **(C)** ARP-1 cells were treated with indicated concentrations of HDS (0, 50 and 100 μM) alone or in the presence of IL-6 or IGF-1 for 72 h. Cell viability was determined by CCK8 assay. **(D)** ARP-1 cells were cultured with or without BMSCs for 72 h in the presence or absence of HDS (0, 50 and 100 μM), and cell growth was assessed using CCK-8 assay. **(E)** ARP-1 cells were exposed to various concentrations of HDS for indicated time. Cell apoptosis was determined by Annexin V/PI staining. Representative results of triplicate experiments are shown (left panel). Apoptotic cells were quantified on the right panel. **(F)** MM cells were treated with HDS for 48 h. Then levels of apoptosis-related proteins were determined by Western blot. Representative results are presented. **(G)** MM cells were incubated with or without pan-caspase inhibitor Z-VAD-FMK for 1 h and then treated with HDS (0 or 50 μM) for 48 h, followed by assessment of cell apoptosis using Annexin V/PI staining. Columns (right panel) represent the average percent of Annexin V positive cells. Data are presented as the means ± SD of 3 independent experiments. **P* < 0.05, ***P* < 0.01.

Supplementary Figure S2


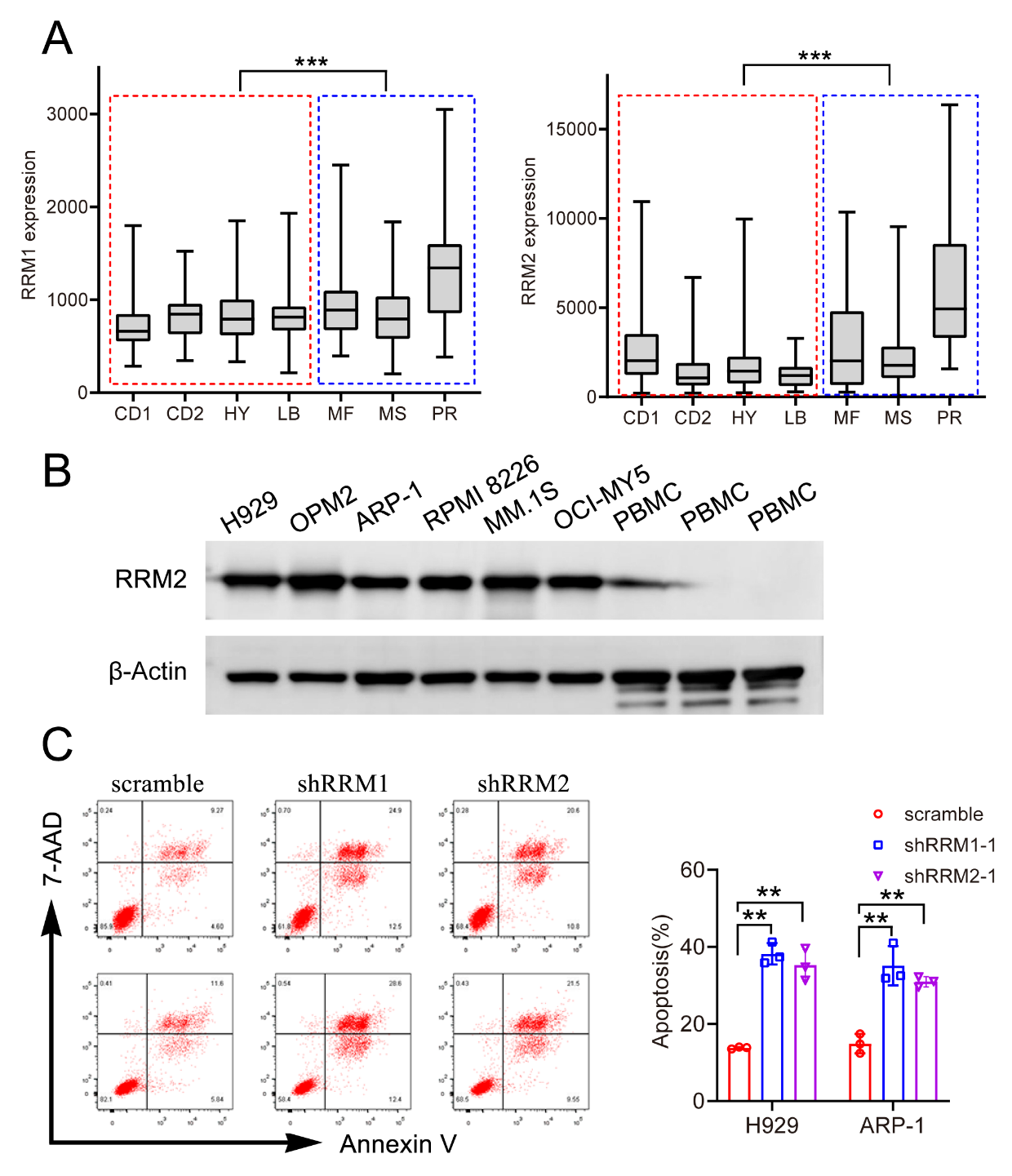


**Supplementary Fig. S2. The oncogenic roles of RRM1 and RRM2 in MM.** **(A)** RRM1and RRM2 expression in plasma cells from patients with MM of Arkansas dataset (GSE4581). Increased RRM1 or RRM2 expression is observed in high-risk subgroups (MF, MS, PR) compared with low-risk (CD1, CD2, HY, LB) subgroups. **(B)** Immunoblot analysis of RRM2 in multiple myeloma cell lines and PBMCs from normal donors. **(C)** Cell apoptosis determined by Annexin V/7-AAD staining after H929 and ARP-1cells was transfected with RRM1-shRNA or RRM2-shRNA. Representative results of triplicate experiments are shown. Data are presented as means ±SD of three independent experiments. ***P* < 0.01, ****P* < 0.001.

Supplementary Figure S3
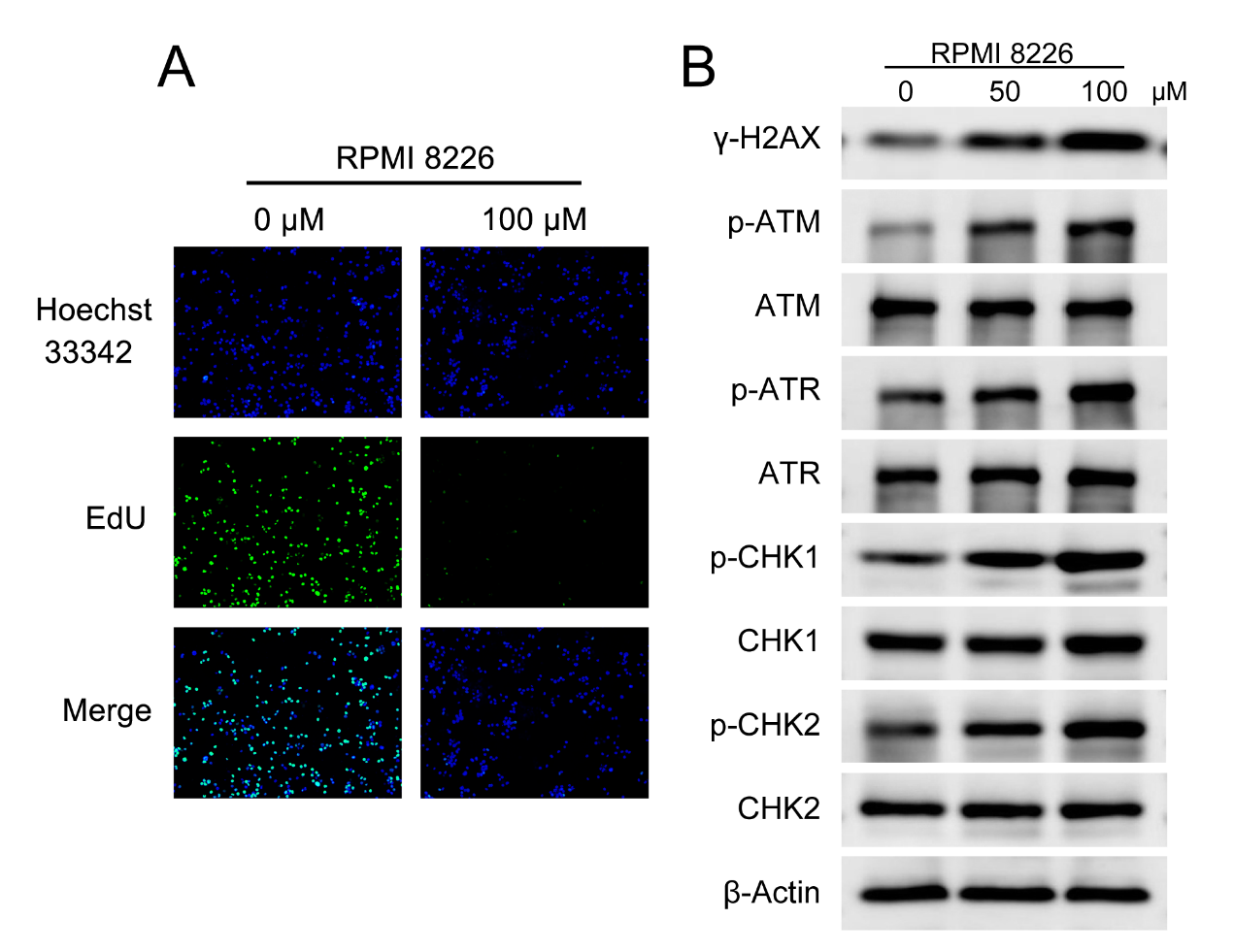


**Supplementary Fig. S3. HDS impaired DNA damage repair by inhibiting dNTP synthesis in RPMI 8226 cells.** **(A)** DNA synthesis in RPMI 8226 MM cells after HDS treatment for 24 h was evaluated by EdU incorporation. **(B)** Western blot analysis of DNA damage-related proteins in RPMI 8226 cells treated with indicated concentrations of HDS for 24 h.

Supplementary Figure S4
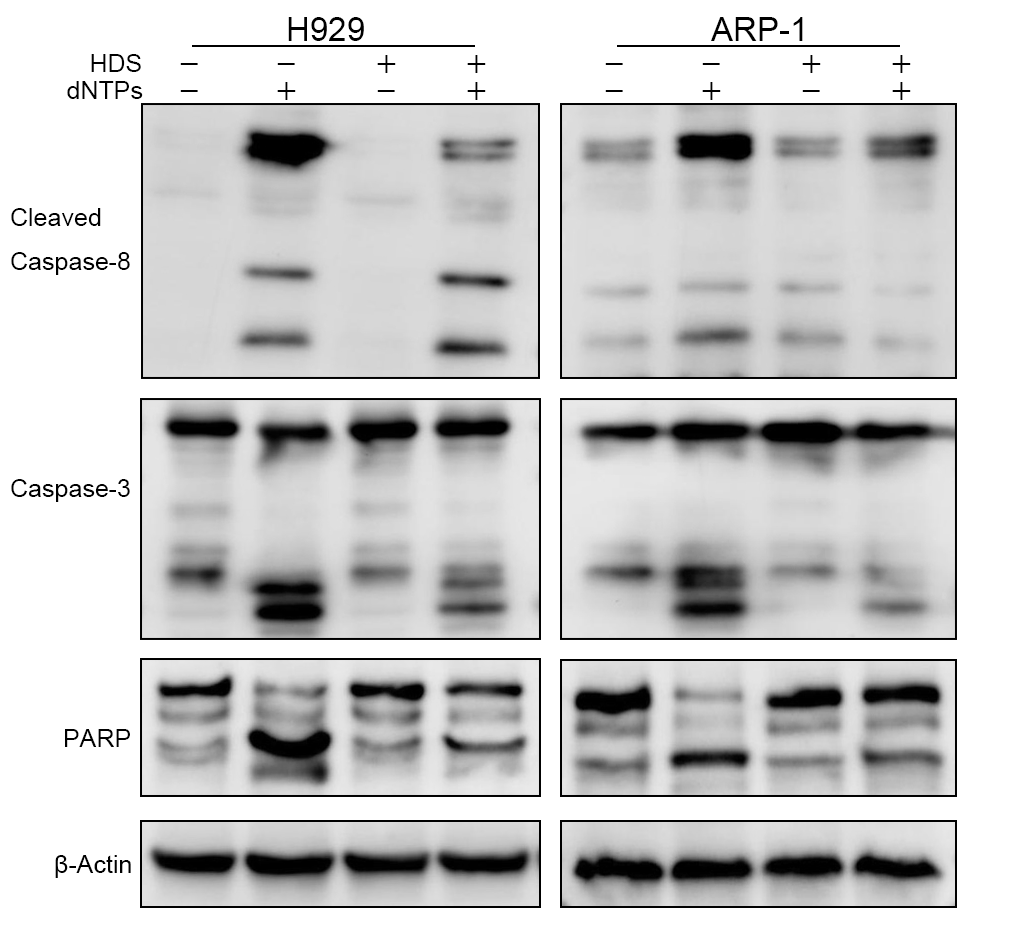


**Supplementary Fig. S4. Exogenous dNTPs relieved HDS induced apoptosis.** H929 and ARP-1 cells were incubated with vehicle, 100 μM HDS, either without or with exogenous 50 μM dNTPs. Apoptosis-related protein expression levels of cleaved caspase-8, caspase-3, and PARP were analyzed by western blot analysis, with β-Actin used as a loading control.

Supplementary Figure S5
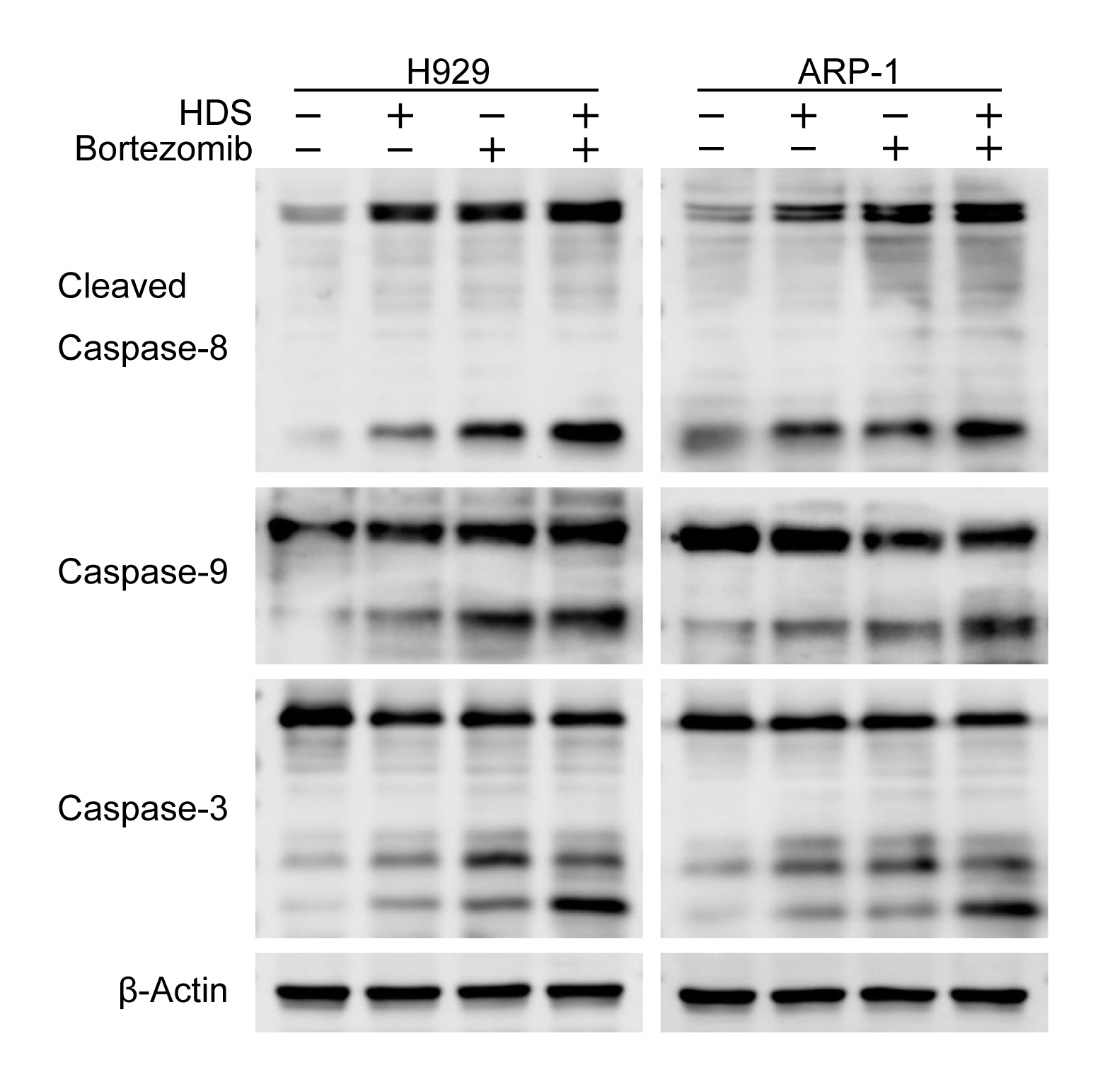


**Supplementary Fig. S5. Synergistic effect of HDS with bortezomib.** The expression of apoptosis-related proteins in MM cells detected by western blot after HDS (50 μM) or bortezomib (10 nM) treatment alone or in combination for 24 h.

Supplementary Figure S6


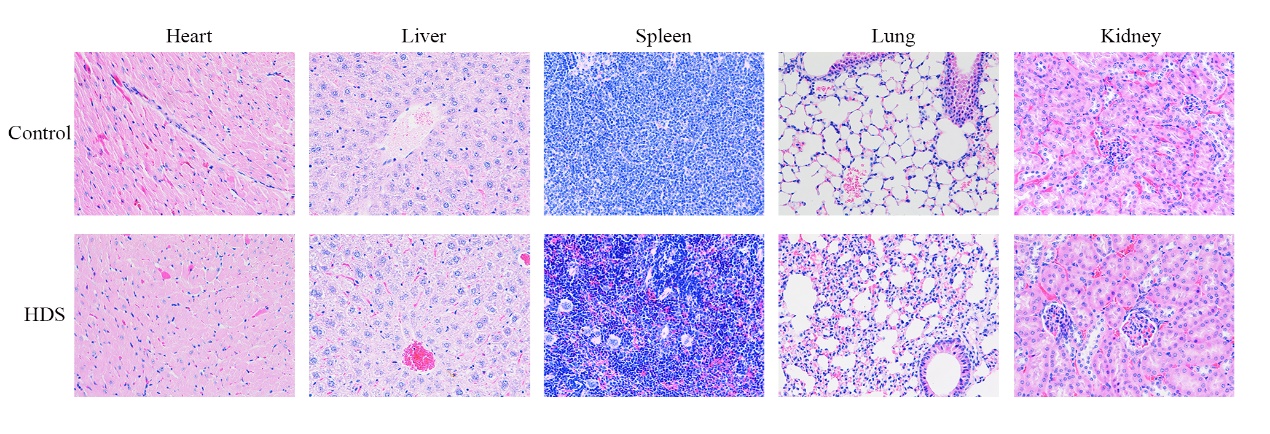


**Supplementary Fig. S6. Effect of HDS on mice tissue.** Results of H&E staining for heart, liver, spleen, lung and kidney of nude mice after 20 days’ treatment with vehicle or HDS. Supplementary Table. S1

**Supplementary Table. S1. The characteristics of MM patients associated Fig 1C.**

| Patient | Sex | Age | Cytogenetics | Last bortezomib-containing therapy | Prior lines of therapy |
| --- | --- | --- | --- | --- | --- |
| 5 | Male | 75 | 1q21  t (4; 14) | Vel/Dex | 2 |
| 6 | Female | 58 | Unknow | VRD | 3 |
| 7 | Female | 51 | del 1p | VCD | 3 |
| 8 | Female | 61 | 1q21 | VRD | 2 |
| 9 | Male | 76 | Unknow | Vel/Dex | 3 |

Vel: bortezomib; Dex: dexamethasone; VRD: bortezomib/ lenalidomide/ dexamethasone; VCD: bortezomib/ cyclophosphamide/ dexamethasone.Supplementary Table. S2


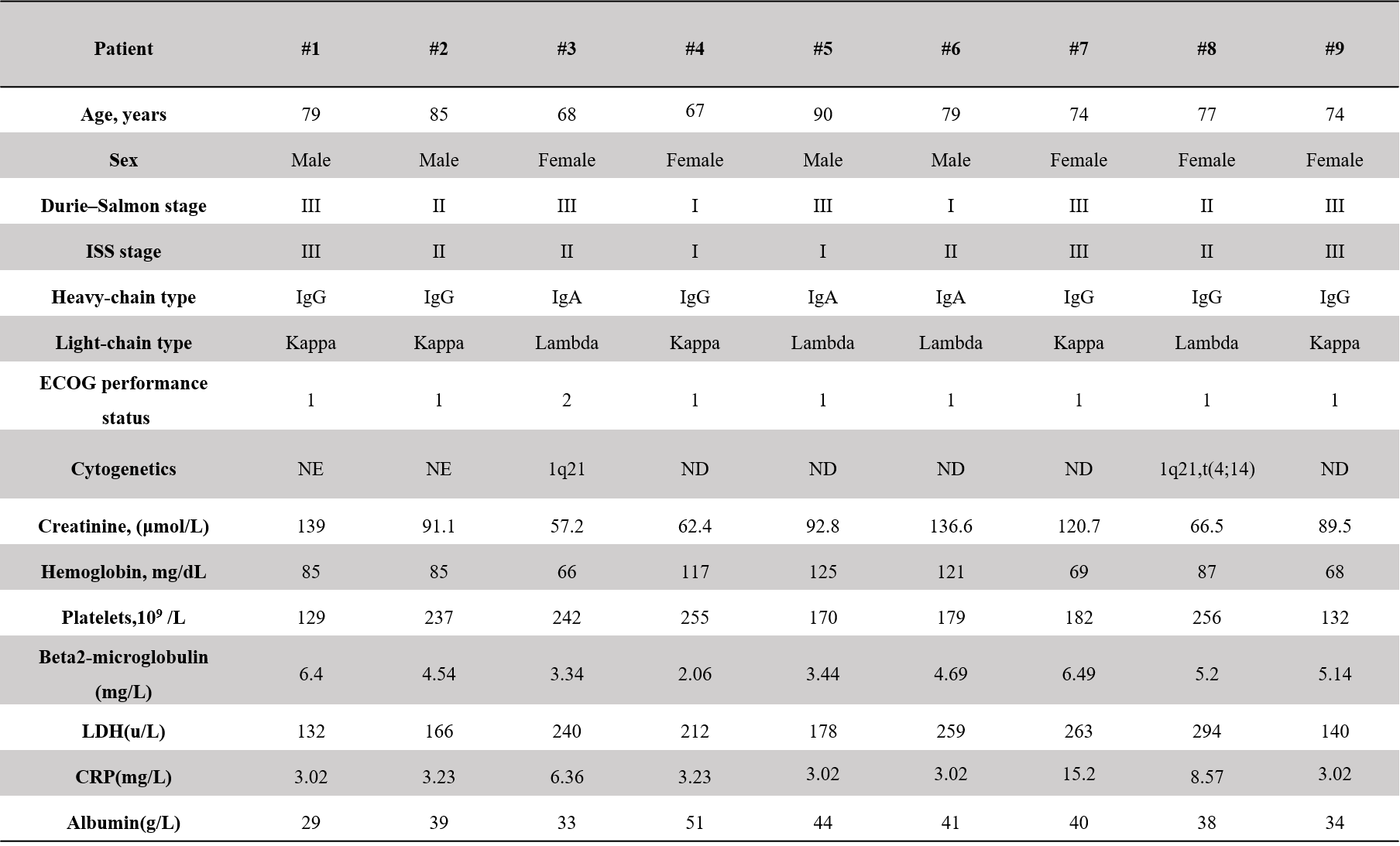


**Supplementary Table. S2. Baseline characteristics of MM patients.** Abbreviations: ECOG, eastern cooperative oncology group; Cytogenetics analysis include Del(13q), Del(17p), t(4;14)，t(11; 14)，t(14;16) and 1q21. NE, negative; ND, not detected.
